# Formation of Magnesium Carbonates on Earth and Implications for Mars

**DOI:** 10.1029/2021je006828

**Published:** 2021-06-22

**Authors:** Eva L. Scheller, Carl Swindle, John Grotzinger, Holly Barnhart, Surjyendu Bhattacharjee, Bethany L. Ehlmann, Ken Farley, Woodward W. Fischer, Rebecca Greenberger, Miquela Ingalls, Peter E. Martin, Daniela Osorio-Rodriguez, Ben P. Smith

**Affiliations:** 1 Division of Geological and Planetary Sciences, California Institute of Technology, Pasadena, CA, USA,; 2 Jet Propulsion Laboratory, California Institute of Technology, Pasadena, CA, USA,; 3 Department of Geosciences, Pennsylvania State University, State College, PA, USA,; 4 Geological Sciences Department, University of Colorado Boulder, Boulder, CO, USA

## Abstract

Magnesium carbonates have been identified within the landing site of the Perseverance rover mission. This study reviews terrestrial analog environments and textural, mineral assemblage, isotopic, and elemental analyses that have been applied to establish formation conditions of magnesium carbonates. Magnesium carbonates form in five distinct settings: ultramafic rock-hosted veins, the matrix of carbonated peridotite, nodules in soil, alkaline lake, and playa deposits, and as diagenetic replacements within lime—and dolostones. Dominant textures include fine-grained or microcrystalline veins, nodules, and crusts. Microbial influences on formation are recorded in thrombolites, stromatolites, crinkly, and pustular laminites, spheroids, and filamentous microstructures. Mineral assemblages, fluid inclusions, and carbon, oxygen, magnesium, and clumped isotopes of carbon and oxygen have been used to determine the sources of carbon, magnesium, and fluid for magnesium carbonates as well as their temperatures of formation. Isotopic signatures in ultramafic rock-hosted magnesium carbonates reveal that they form by either low-temperature meteoric water infiltration and alteration, hydrothermal alteration, or metamorphic processes. Isotopic compositions of lacustrine magnesium carbonate record precipitation from lake water, evaporation processes, and ambient formation temperatures. Assessment of these features with similar analytical techniques applied to returned Martian samples can establish whether carbonates on ancient Mars were formed at high or low temperature conditions in the surface or subsurface through abiotic or biotic processes. The timing of carbonate formation processes could be constrained by ^147^Sm-^143^Nd isochron, U-Pb concordia, ^207^Pb-^206^Pb isochron radiometric dating as well as ^3^He, ^21^Ne, ^22^Ne, or ^36^Ar surface exposure dating of returned Martian magnesium carbonate samples.

## Plain Language Summary

Magnesium carbonate minerals rarely form large deposits on Earth and because they constitute such a small proportion of the terrestrial carbonate record in comparison to calcium-rich carbonates, they have received little attention. In contrast, the largest carbonate deposit detected on Mars has magnesium carbonate, and it has been detected at the landing site of the 2020 mission where the Perseverance rover will collect samples for return to Earth. We synthesized the field observations and laboratory experiments that pertain to magnesium carbonates formed on Earth and find that they form in five types of environments as follows: within veins or in the bulk volume of magnesium-rich rocks, soils, alkaline or salty lakes, and as replacements of previously formed calcium-rich carbonate minerals. Conceptually, these environments may be analogs for ancient Martian magnesium carbonate-forming environments. Magnesium carbonates formed in some environments are capable of preserving remnants of microbes, especially if magnesium carbonates formed characteristic large-scale clotted or vertical column shapes or microscale spherical and laminated textures. If life had ever originated on Mars, these would be key materials to investigate for biosignatures. Finally, a number of analytical techniques are discussed that can be performed on magnesium carbonate rocks collected by Perseverance when returned to Earth.

## 1. Introduction: Magnesium Carbonates on Earth and Mars

The Perseverance rover will explore and sample, for eventual return to Earth, magnesium carbonates found at its Jezero crater landing site on Mars (Grant et al., 2018), motivating interest in understanding the possible formation pathways of these uncommon carbonate minerals. Terrestrial magnesium carbonate minerals include a range of anhydrous and hydrous phases. Magnesite (MgCO_3_) is the Mg-rich endmember of carbonate minerals with a trigonal crystal structure. It forms solid-solution series and cooccurs with corresponding calcium- and iron-rich carbonate endmembers. Although in low-temperature surface environments, the stability of the solid solution series is highly affected by kinetic effects (Chai & Navrotsky, 1996; Morse & Mackenzie, 1990). Hydrous magnesium carbonate mineral phases, such as hydromagnesite (Mg_5_(CO_3_)_4_(OH)_2_·4H_2_O), barringtonite (MgCO_3_·2H_2_O), nesquehonite (Mg(HCO_3_) (OH) ·2(H_2_O)), lansfordite (MgCO_3_·5H_2_O), artinite (Mg_2_(CO_3_) (OH)_2_·3H_2_O), and dypingite (Mg_5_(CO_3_)4(OH)_2_·5(H_2_O)), often cooccur with magnesite (e.g., Power et al., 2014; Zedef et al.,. 2000).

On Earth, the Ca-rich carbonate endmembers such as calcite (CaCO_3_; trigonal), aragonite (CaCO_3_; orthorhombic), and dolomite (CaMg(CO_3_)_2_; trigonal) are far more abundant than either magnesite or siderite (FeCO_3_). Atmospheric carbon dioxide produces carbonate rock through aqueous chemical weathering. Such reactions are central to the surficial carbon cycle and rock cycle on Earth. The dominance of calcium carbonates results from the fact that modern seawater is oversaturated with calcium carbonate due to weathering of Ca-dominant silicate rocks (Walker et al., 1981) and preexisting carbonate rocks. Ca appears to have dominated for even the earliest part of Earth history that preserves a sedimentary rock record (Grotzinger & James, 2000; Grotzinger & Kasting, 1993). Since marine sediments dominate Earth’s stratigraphic record, deposits of calcium carbonate overwhelm the tiny proportion of primary magnesium carbonate precipitation on Earth.

In contrast, deposits with magnesium carbonate minerals reveal clues about past aqueous activity on the Martian surface (e.g., Edwards & Ehlmann, 2015; Ehlmann et al., 2008; McSween et al., 2015). Although Mars is currently too dry for detectable large-scale aqueous alteration at its surface, mineralogical evidence for hydrated minerals and geomorphological evidence of fluvial, glacial, and lacustrine systems indicate abundant surface waters prior to about 3.5 Ga (Carr, 1987; Hynek et al., 2010). One candidate mechanism proposed for the dramatic climatic cooling on Mars is drawdown of atmospheric CO_2_ into carbonate rock (Kahn, 1985). However, despite evidence for a wetter past and the current composition of the atmosphere of ~95% CO_2_, surficial carbonate rock on Mars is remarkably rare. On Mars, orbital spectroscopy reveals widespread hydrated mineral phases, indicating aqueous alteration (Bibring et al., 2006; Mustard et al., 2008), including phyllosilicates, sulfates, and hydrated silica, but only limited surface exposure of carbonates (Ehlmann et al., 2008; Niles et al., 2013). Bandfield (2003) detected magnesium carbonate at the few wt% level in globally widespread Martian dust, and Boynton et al. (2009) measured 3–5 wt.% calcium carbonate at the Phoenix landing site. Curiosity X-ray diffraction data and evolved gas analysis data indicate ~1 wt. % Mg, Fe carbonate in soils (Archer et al., 2020; Leshin et al., 2013). Mg-Fe carbonates were also observed at ~25 wt. % in a small, olivine/silica-rich volcaniclastic outcrop at the Spirit landing site (Morris et al., 2010). Carbonates also occur in some Martian meteorites (Niles et al. 2013), including the nodules consisting of mixtures of Mg-, Fe-, and Ca-rich carbonates in ALH84001 (Valley, 1997).

**Figure 1.**
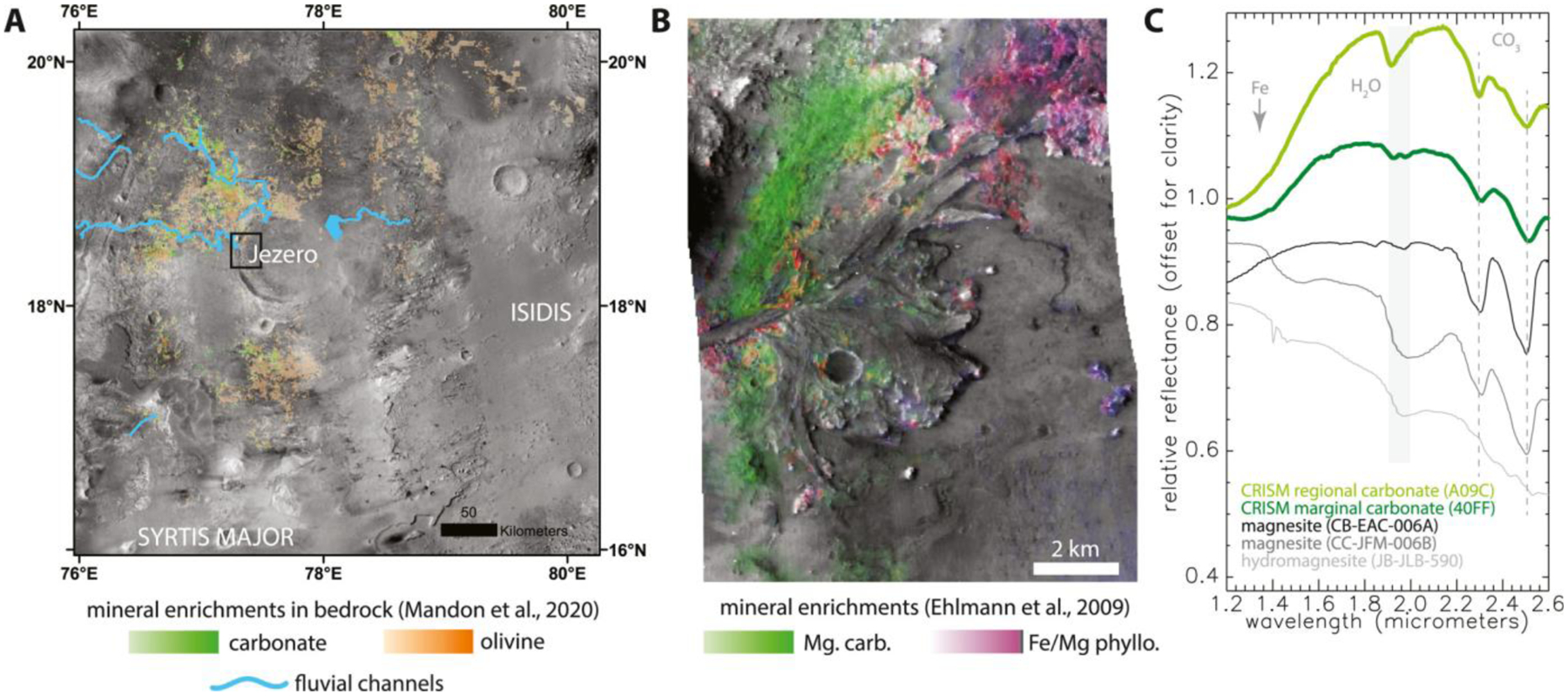
(a) The watershed of Jezero crater lake cross-cuts olivine-enriched bedrock, in places altered to magnesium carbonate. Map of bedrock enrichments, subsetted from Mandon et al. (2020). (b) The sediments of the western shore and delta fed by the western channel are enriched in magnesium carbonate (green), relative to the sedimentary rocks deposited by the northern channel. Map of mineral enrichments, subsetted from Ehlmann et al., (2009). (c) Infrared spectra acquired of rock units from orbit compared with spectra from the RELAB spectral library. Note: characteristic 2.3 and 2.5 micrometer absorptions associated with Mg bound to CO_3_ and 1.9 micrometer absorptions associated with H_2_O.

From orbital remote sensing, a single region of Mars (Nili Fossae) accounts for the majority of the areal fraction carbonate detections known for Mars (Wray et al., 2016; Ehlmann et al., 2008) (Figure 1). A few dozen small-scattered locales in the southern highlands have infrared signatures consistent with a mineral assemblage of smectite clay, chlorite, and Fe/Ca carbonate (Wray et al., 2016). The Nili Fossae carbonate is associated with an olivine-bearing unit modeled to contain 10%–40% coarse-grained olivine and up to 20 wt.% magnesium carbonate with minor amounts of Fe/Mg phyllosilicate along with pyroxene and feldspar (Edwards & Ehlmann, 2015; Hoefen et al., 2003; Mandon et al., 2020; Salvatore et al., 2018) (Figure 1a). Ehlmann et al. (2008) concluded that the dominant cation in the carbonate is Mg, due to infrared absorption positions distinct from Ca and Fe carbonates (Figure 1c) (Gaffey, 1987). The spectra additionally exhibit an H_2_O absorption, particularly in the regional bedrock (Figure 1c) (e.g., Ehlmann et al., 2008). Some terrestrial magnesium carbonates that are nominally anhydrous (e.g., magnesite) can exhibit H_2_O absorptions (e.g., Gaffey, 1985; Leask, 2020); such absorptions are also characteristic of hydrous carbonates like hydromagnesite (Horgan et al., 2020). The presence of these carbonates could reflect alteration of the olivine unit involving surface water, shallow groundwater, or deeper hydrothermal solutions. In the primary mission, the Perseverance rover is expected to examine magnesium carbonate within crater floor units and deltaic sedimentary rocks in the Jezero crater paleolake basin (Figure 1b). In an extended mission, the rover may examine magnesium carbonate in situ within the regional olivine unit.

The Mars 2020 mission is tasked with seeking the signs of life in ancient Martian environments, understanding the geological history of Mars, and preparing a collection of samples for Earth return by the future Mars Sample Return program (Williford et al., 2018). The carbonates at Jezero crater and the surrounding Nili Fossae region are key lithologies to be investigated and sampled by the Perseverance rover. Thus, the sampling efforts and subsequent analyses of Martian magnesium carbonates require an understanding of processes that form magnesium carbonates on Earth. This synthesis reviews the geological environments and physical conditions in which magnesium carbonates form on Earth in order to constrain pathways for magnesium carbonate formation in ancient Martian environments, the potential preservation of biosignatures, and the use of stable isotopic, elemental, and geochronologic systematics in their evaluation.

For this purpose, we divide the study into sections that review the following topics: (a) Magnesium carbonate-forming environments on Earth. (b) Characteristic textures and fabrics found within different carbonate-forming environments on Earth and considerations of how these can be tied to interpreting formation environments. (c) Thermodynamic and kinetic conditions required to precipitate magnesium carbonates in synthetic experiments and how this can help constrain magnesium carbonate formation on Mars. (d) Results from isotopic and elemental chemistry laboratory methods, and how application of these methodologies to Martian samples can aid in interpretation of formation conditions. (e) Geochronological methods that have been applied to magnesium carbonates for geochronological study of returned samples of Martian carbonates.

## 2. Geological Context of Magnesium Carbonate Formation

**Figure 2.**
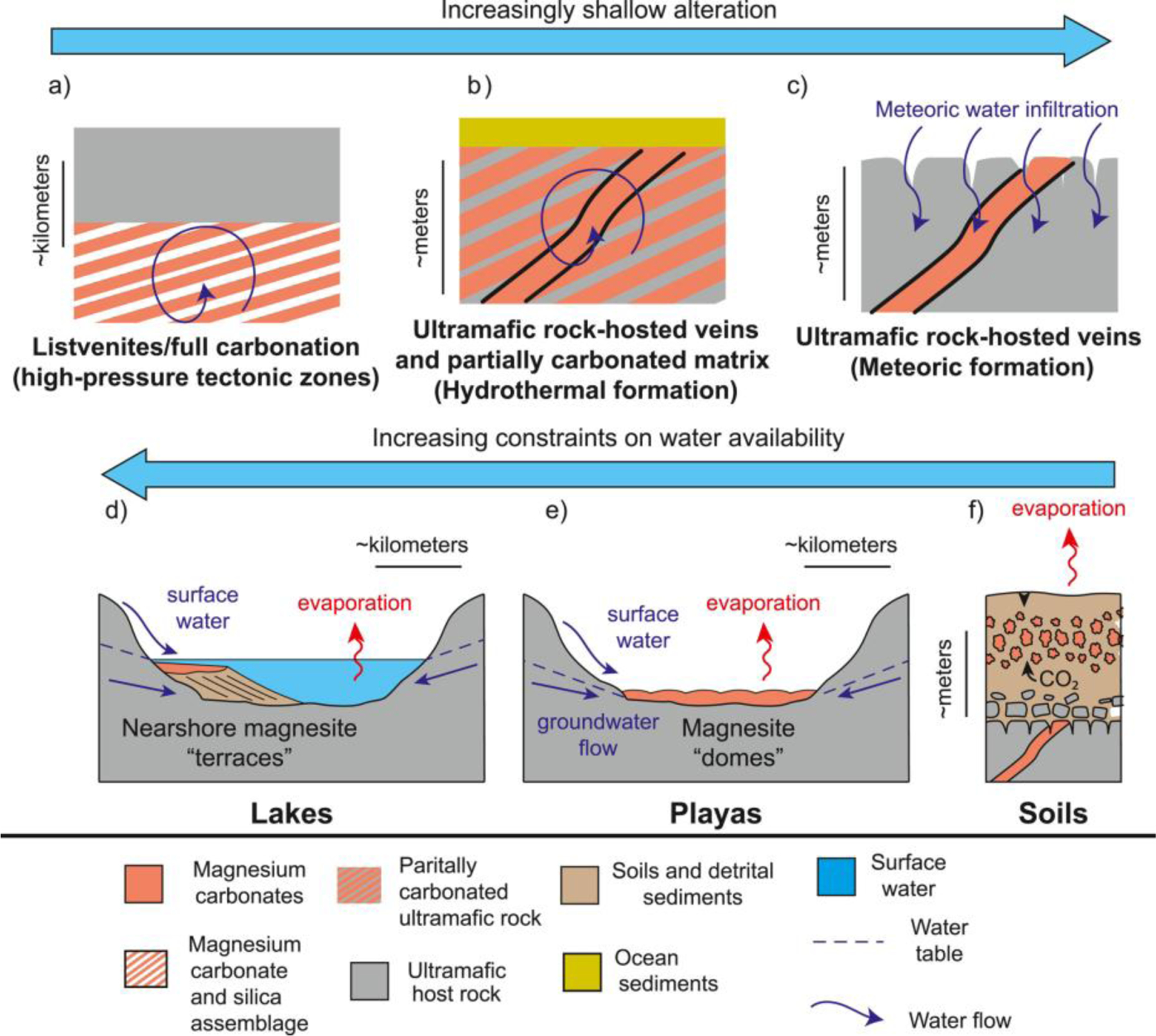
Schematic depicting the three main ultramafic rock-hosted and three main sedimentary magnesium carbonate-forming environments on Earth. (a) magnesium carbonate and silica assemblage as alteration products in fully carbonated peridotite within subduction zones known as listvenite, magnesium carbonates as veins in ultramafic host rock formed by (b) hydrothermal alteration or (c) meteoric water infiltration, (d) magnesium carbonate terraces/stromatolites within alkaline lakes, (e) magnesium carbonate crusts in playas, and (f) magnesium carbonate nodules in soils in close association with ultramafic rock-hosted magnesite veins.

Magnesium carbonates on Earth (Figure 2) are commonly associated with ultramafic rocks, either in (1) veins or (2) within the matrix of carbonated peridotite or, often through transport of fluids that have interacted with ultramafic rock, as authigenic precipitates, that is, (3) nodules in soil, and in (4) alkaline lakes and playas. A fifth occurrence (5) is within diagenetically or hydrothermally altered limestones and dolostones.

Magnesite can also form within the upper mantle by transformation of subducting dolomite, and they persist to the lower mantle (Isshiki et al., 2004). However, since these mantle occurrences are not related to surface processes, we do not discuss them further. Magnesite and other products from the magnesite-siderite solid solution series can be found within igneous rocks that have a mineralogical composition of >50% carbonate minerals, also known as carbonatites (Buckley & Woolley, 1990; Zaitsev et al., 2004). Although some phases of magnesite in carbonatites have been suggested to be potential primary liquidus phases based on their characteristic euhedral crystal shape (Buckley & Woolley, 1990), experimental data suggest that magnesite can only crystallize at mantle-like pressure/depths (>20 Kb) from carbonate melts, which much more typically form Ca-rich carbonates (Irving & Wyllie, 1975; Lee & Wyllie, 2000). Instead, the carbonatite-associated magnesites formed through hydrothermal alteration or as replacements of other carbonates akin to alteration processes found within ultramafic and sedimentary carbonate rocks, described in the following sections (Buckley and Woolley, 1990; Zaitsev et al., 2004).

The primary five geological settings provide viable Earth analogs for the magnesium carbonate detections localized in Jezero crater and its adjacent highlands on Mars. The geologic context and textures of magnesium carbonates observed by the Perseverance rover will help determine the environmental setting of magnesium carbonate formation on ancient Mars inside and outside Jezero crater. Descriptions of magnesium carbonate systems in this section are separated into ultramafic rock-hosted, sedimentary, and impact-associated systems on Earth. Additional diagenetic, metasomatic, and metamorphic replacement environments are described in Section 3. Finally, the section summarizes Martian occurrences of magnesium carbonates in detail for comparison with terrestrial analogue environments.

### 2.1. Ultramafic Rock-Hosted Magnesium Carbonate on Earth

Magnesite is found filling fractures in ultramafic rocks on Earth, particularly within ophiolites, where the ultramafic oceanic crust and upper mantle have been tectonically thrust onto the preexisting continental margin (e.g., Abu-Jaber & Kimberley, 1992; Schroll, 2002). In fact, magnesite is present in most ophiolites. Veins, carbonated peridotite matrix, and overlying pedogenic nodule-type magnesium carbonates within ultramafic rock record a range of aqueous environments and conditions ranging from deep-seated to shallow hydrothermal activity to infiltration and alteration by low-temperature meteoric fluids via fractures to low-temperature laterization and surface weathering. These give rise to characteristic and distinguishable textures such as veins, nodules, and matrix magnesium carbonates within carbonated ultramafic rock. A record of one and possibly multiple transitions of such environments on Mars would therefore yield important insights into the temperature, chemistry, and nature (surface vs. subsurface) of ancient aqueous activity on Mars.

#### 2.1.1. Hydrothermal Processes Leading to Complete and Partial Carbonation

A continuum exists between magnesium carbonate formation hydrothermally and by chemical weathering. The most common terrestrial setting for hydrothermal magnesium carbonate formation is in ultramafic rocks within ophiolite deposits. Here, a common pathway is first the serpentinization of the ultramafic rock and later the carbonation of the serpentinite (Klein & Garrido, 2011; Klein & McCollom, 2013), though the particular fluid chemistries and CO_2_ availabilities determine whether serpentine or magnesite is favored. Full carbonation occurs when primary pyroxene and olivine have been completely altered and replaced by a distinct carbonate and quartz assemblage with new textures compared with the former peridotite (e.g., Falk & Kelemen, 2015; Halls & Zhao, 1995; Nasir et al., 2007). This fully carbonated quartz-carbonate rock forms via a set of dissolution-reprecipitation reactions and is called listvenite (or listwaenite). Full carbonation to listvenite typically requires high pressures of ~0.2–1.5 GPa, provided by slab subduction, which increases the solubility of CO_2_ in fluids by orders of magnitude (Falk and Kelemen, 2015; García del Real & Vishal, 2016; Kelemen and Manning, 2015). Listvenites within the Advocate ophiolite, Newfoundland, Canada, Birjand ophiolite, Iran, and Semail ophiolites are all interpreted to have formed through carbonation by fluids from the subducting slab (Boskabadi et al., 2020; Menzel et al., 2018). These terrestrial listvenite-forming geologic environments form in high-pressure tectonic environments with large CO_2_ sources, the combination of which is unlikely on the ancient Martian surface, which was not tectonically active (Nimmo & Stevenson, 2000). High pressures during meteorite impacts are likely to be too transient or would lead to melting (see Section 2.3).

However, the solubility of CO_2_ is a function of temperature, pressure, and salinity among other factors (e.g., Duan & Sun, 2003), and carbonation can also occur at lower pressures. For example, incomplete carbonation during hydrothermal alteration forms a large array of mineral assemblages at much lower pressures that are plausible for Mars (<0.5 GPa; Grozeva et al., 2017; Klein & Garrido, 2011; Klein & McCollom, 2013), including but not limited to the following: (a) serpentine + brucite + magnesite, (b) serpentine + magnesite, (c) serpentine + talc + magnesite, (d) talc + magnesite, or soapstone (Beinlich et al., 2012; Hansen et al., 2005; Menzel et al., 2018), as well as assemblages containing magnetite, goethite, hematite, and metal alloys (e.g., Kelemen et al., 2011). Magnesium carbonates in these rock types form veins (e.g., Ashley, 1997; Boschi et al., 2009), and also occur as part of the matrix assemblage of the ultramafic rock, typically with microcrystalline textures (Beinlich et al., 2012; Hansen et al., 2005; Menzel et al., 2018). While discussion of the full pathway from dunite or harzburgite through intermediates to listvenite is beyond the scope of this magnesite review, we note that other minerals that cooccur with magnesite provide key information about the temperature, pressure, fluid chemistry, and redox conditions of the alteration. The complete reactions involved in the carbonation process are the subject of ongoing study (e.g., by the recent Oman Drilling Project; Kelemen et al., 2020).

Although hydrothermal magnesite occurrences in ultramafic complexes are much more common, other settings with mafic rocks do occur. For example, magnesium carbonates are found as hydrothermal alteration products in basalts within deposits in Northern Spitsbergen, Svalbard (Treiman et al., 2002). Here, they form as relatively minor precipitates in veins and may be related to alteration of the ultramafic xenoliths within the lavas.

#### 2.1.2. Chemical Weathering Processes

At lower temperatures and in waters in contact with atmosphere, chemical weathering processes at more moderate temperatures alter ultramafic rocks. Olivine and pyroxene in the ultramafic rock are Mg-rich and relatively Si-poor and Ca-poor. Fluids become alkaline as they react with the bedrock, and in the presence of atmospheric CO_2_, Mg^2+^- and HCO_3_^−^-bearing fluids form from chemical weathering (e.g., Barnes & O’Neil, 1969; Neal & Stanger, 1985). These fluids tend to precipitate magnesite and occur as surface waters and shallow ground waters (e.g., Barnes & O’Neil, 1969; Kelemen et al., 2011). Upon infiltrations, the fluids change composition as dissolved CO_2_ is exhausted via Mg carbonate precipitation. During low temperature serpentinization by these alkaline fluids, now CO_2_-poor waters, pyroxene alteration by hydration that produces serpentine releases Ca^2+^ (from pyroxene) and generates OH^−^, yielding Ca^2+^- and OH^−^-rich waters (Barnes & O’Neil, 1969; Kelemen & Matter, 2008; Kelemen et al., 2011; Neal & Stanger, 1985). These waters precipitate calcium carbonates upon renewed contact with the atmosphere and, thus, travertine springs are a common feature of serpentinized ultramafic bodies.

**Figure 3.**
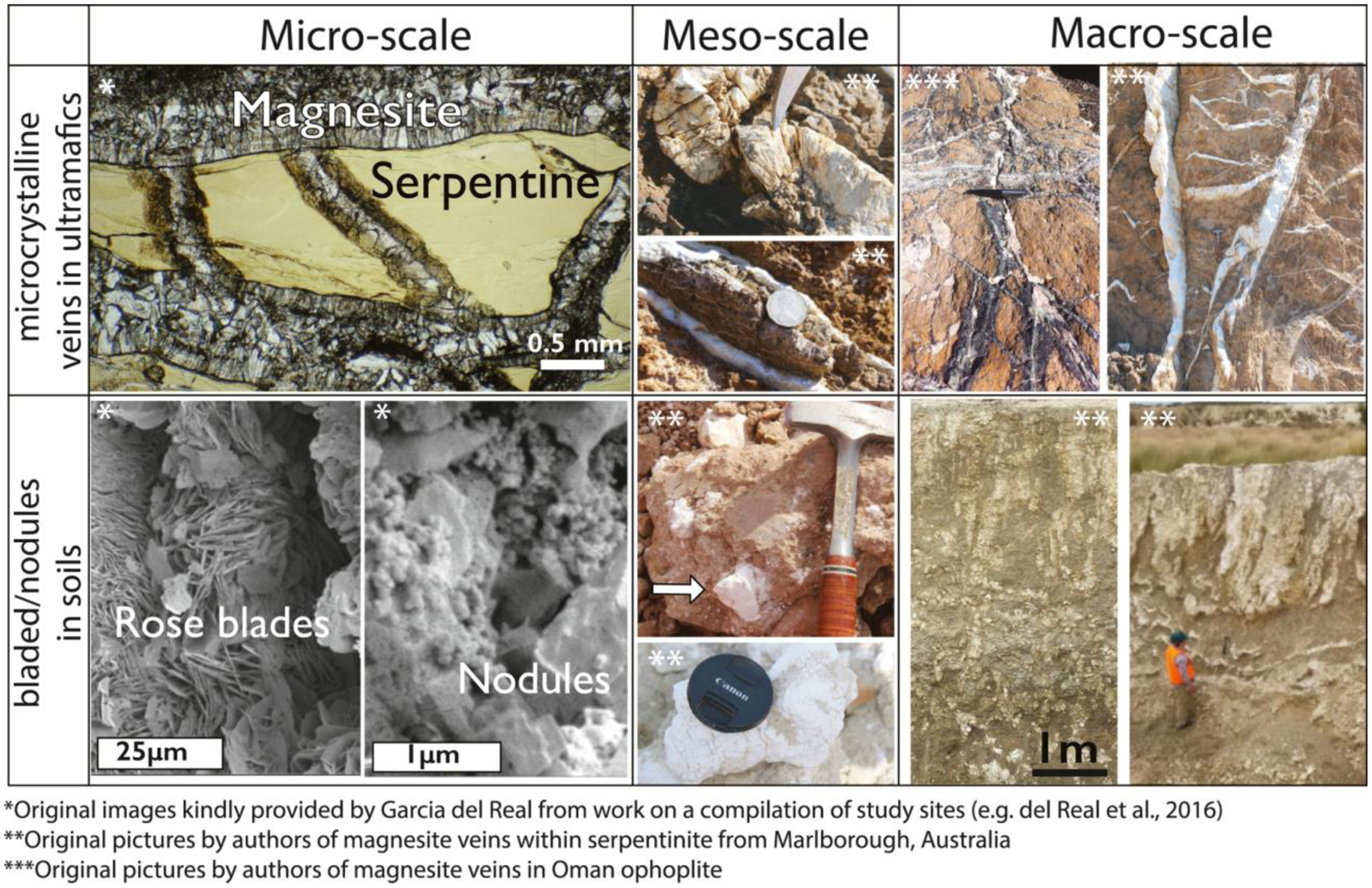
Key textures at micro-, meso-, and macro-scale in ultramafic terrains (upper row) and pedogenic soils overlying ultramafic terrains (lower row). Full length of hammer for scale is ~32 cm. Quarter for scale is ~2.5 cm. Pen for scale is ~10 cm. Lens cap for scale is ~5 cm.

The Semail ophiolite of Oman and the United Arab Emirates hosts an 8–12 km thick sequence of particularly well studied ultramafic rock, upper mantle peridotite (e.g., Glennie et al., 1973; Searle & Cox, 1999). Magnesite is common within the ophiolite, occurring in veins that are up to a few meters thick (Figure 3) (e.g., Kelemen & Matter, 2008; Kelemen et al., 2011; Mervine et al., 2014; Neal & Stanger, 1985). Neal and Stanger (1985) estimate that 80% of carbonate veins within the mantle rock of the ophiolite are magnesite. Hydromagnesite, nesquehonite, and dypingite and other related minerals (e.g., aragonite, brucite, etc.) are also present as rock coatings and crusts within spring deposits (e.g., Giampouras et al., 2020). Magnesite vein outcrops are separated spatially from surface-outcropping travertines (Ca-carbonates), which formed from discharging alkaline Ca^2+^- and OH^−^-rich waters (Clark & Fontes, 1990; Kelemen et al., 2011). While the ophiolite obducted >90 Ma (Rioux et al., 2012, 2013, 2016), radiocarbon can be readily detected in carbonate veins, and dating yields ~8–45 kyr ages for most veins (Kelemen et al., 2011; Mervine et al., 2014; see Section 6). This suggests that magnesite veins are actively forming through low temperature surface weathering (Kelemen et al., 2011).

Similarly, deep veins within the New Caledonia ophiolite contain magnesite and are interpreted to form through alteration by meteoric water, possibly associated with laterization at the surface (Figure 3) (Quesnel et al., 2013, 2016; Ulrich et al., 2014). Magnesite veins up to 10-m thick within ophiolite blocks in NE Iran, such as the Derakht-Senjed deposit, are associated with the Mg-rich, Ca-poor fluids, indicative of weathering processes (Mirnejad et al., 2008, 2015). The Attunga magnesite deposit within the Great Serpentinite Belt deposit contains veins and nodules within the ultramafic rock interpreted to have formed through weathering (e.g., Oskierski et al., 2013). Furthermore, characteristic vein and stockwork magnesite have been identified in the Dinarides in the Balkan Peninsula (referred to as Yugoslavia in past literature) (Fallick et al., 1991; Jurković et al., 2012). However, studies disagree on whether these deposits originated through a hydrothermal or weathering process.

These settings of chemical weathering of ultramafic rocks were suggested as an original analog for the Mg carbonates on Mars (Ehlmann et al., 2008) because cold temperature weathering under water-limited conditions will tend to promote magnesium carbonates but not the later stages of serpentine or calcium carbonate formation, which is common in the water-rich terrestrial systems but not observed in associated with the deposits on Mars.

#### 2.1.3. Magnesite Precipitation During Soil Formation

Magnesium carbonates are also associated with the soils in ultramafic regions and represent a combination of chemical weathering as well as dissolution-reprecipitation reactions as meteoric fluids and organic acids from vegetation interact with ultramafic rocks. In addition to deep veins, the ophiolites in New Caledonia and Attunga, Australia, are also associated with pedogenic environments directly overlying ultramafics, and are interpreted to represent surface alteration of the ultramafics (Oskierski et al., 2013; Quesnel et al., 2013, 2016). These pedogenic soils in New Caledonia and Attunga often contain nodular concretions of magnesite with cauliflower textures (Figures 2 and 3). A similar setting is found at the Woodsreef Asbestos Mine in the Great Serpentinite Belt, Australia, where hydromagnesite, magnesite, and other carbonates formed through reaction of meteoric fluids with mine tailings (Oskierski et al., 2013).

### 2.2. Sedimentary Magnesium Carbonate Deposits on Earth

Magnesite-forming systems are often related to hydrologic conditions within broader ultramafic catchments. Permanent lakes form when evaporation is balanced by recharge from the watershed, and playas form when evaporation exceeds recharge, so that the basin is intermittently dry. However, lakes and playas are similar; the dissolved ions are sourced from the surrounding watershed rather than bedrock directly underneath the deposit (Braithwaite & Zedef, 1996; Power et al., 2014). Furthermore, numerical modeling of evaporite basins has shown that even thin (10s of cm) evaporite beds require large volume of water to be flushed through the system, often exceeding the total lake volume by a factor of 1,000 or more (Wood & Sandford, 1990). As such, ancient magnesite from playas or lakes could be qualitative indicators of long-term water availability and/or chemical weathering rates in the surrounding drainage area (Figure 2). In contrast, magnesite-bearing soils (Quesnel et al., 2016; Schroll, 2002) are more difficult to interpret; some soils form from groundwater discharge into topographic lows (Oskierski et al., 2013), but others could be in situ weathering products where ions are sourced directly from underlying bedrock (Figure 2).

**Figure 4.**
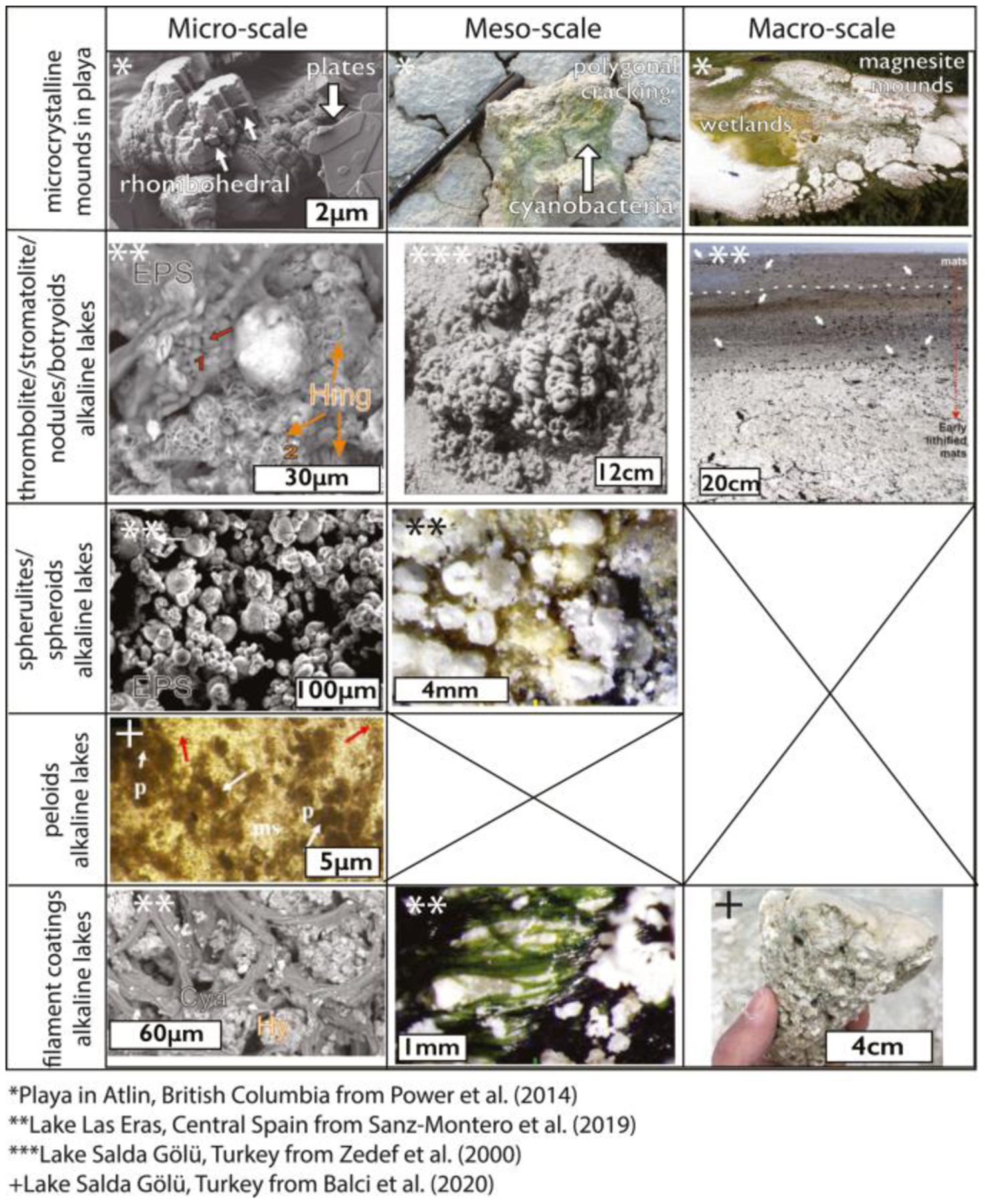
Key textures at micro-, meso-, and macro-scale in hypersaline and alkaline lake environments. White arrows in alkaline lake macroscale indicate location of mat mounds. Playa mounds macroscale image is an aerial photograph and hence missing scale. The symbols denote the source of images from publications or contributed by individuals as listed in the bottom. Hmg = hydromagnesite, EPS = extrapolymeric substances, Cya = cyanobacteria, Hy = hydromagnesite, p = peloid, ms = microsparitic.

Magnesium carbonates have been observed to precipitate in perennial alkaline lakes, ephemeral playas (e.g., Alçiçek, 2009; Braithwaite & Zedef, 1996; Mur & Upinell, 1987), soils (Oskierski et al., 2013; Quesnel et al., 2013; Schroll, 2002), and coastal salinas (von der Borch, 1965) (Figures 2 and 4). All of these lakes and playas are within or adjacent to ultramafic units. For example, Lake Salda, Turkey, contains hydromagnesite mounds, terraces, and stromatolites (Figures 2 and 4) (Braithwaite & Zedef, 1996; Russell et al., 1999; von der Borch, 1965; Zedef et al., 2000). This ~200 m deep lake has waters of pH > 9 (Braithwaite & Zedef, 1996), and Mg was likely sourced through the Lake Salda watershed ground and surface waters interacting with the surrounding serpentinized ultramafic rock (Zedef et al., 2000). Lake Salda has recently been revisited by Horgan et al., (2020) as an analog for the “marginal carbonates” in Jezero crater, Mars, which occupy a restricted elevation range that tracks the elevation of the delta top, suggesting a possible shoreline deposit for those carbonates (Horgan et al., 2020). At Lake Salda, hydromagnesite precipitates as coatings and cements in nearshore sediment associated with rocky shorelines and deltas (Braithwaite & Zedef, 1996). In addition, hydromagnesite comprises stromatolites in Lake Salda, which provides an analog for possible biosignature preservation on Mars (Braithwaite & Zedef, 1996; Russell et al., 1999).

Magnesium carbonate-bearing playas have been well documented near Atlin, British Columbia (Power et al., 2007, 2014, 2019). Regional ophiolites are the likely source of Mg to these playas, and the climate is subarctic with carbonates forming at freezing conditions (Power et al., 2007, 2014, 2019); this last point is significant and means magnesium carbonates can and do form across a wide range of temperature conditions (Bosak et al., 2021). This is relevant to Mars as the Martian surface temperature varies widely (−130°C–30°C) but is generally much cooler than the Earth’s surface (e.g., Vasavada et al., 2017). Magnesium carbonates form low relief mounds (10s of cm) in the center of the playa (Figure 4). These mounds include mixtures of magnesite and metastable magnesium carbonates such as hydromagnesite, nesquehonite, and landsfordite (Power et al., 2014). While magnesite precipitation is initiated subaqueously, mounds in the Atlin playas continued to grow and amalgamate after their emergence from the surrounding ephemeral ponds (Power et al., 2019). Exposure features in these deposits consist of polygonal desiccation cracks and “cauliflower” textures, interpreted to require limited surface water during magnesite formation (Power et al., 2014). In ephemeral systems, magnesite precipitates from playa lakes in Los Monegros, Spain, form the combination of seasonal Mg-rich brines and decaying organic matter increasing CO_2_ (Mur & Urpinell, 1987).

The 2 Ga Tulomozerkskaya Formation in the Fennoscandian Shield, Scandinavia, includes five beds of magnesite interpreted to have formed in a playa or sabkha environment in which micritic magnesite diagenetically replaced dolomite and magnesite precipitated directly from brines (Melezhik et al., 2001). In this Paleoproterozoic example, magnesite replacement appears to have been stimulated by evaporative modification of mixed marine-terrestrial fluids rather than weathering of ultramafic rocks. A modern analog for this type of magnesite precipitation is found in the coastal salinas of the Coorong region of South Australia (von der Borch, 1965; Walter et al., 1973). In the ~790 Ma Skillogalee Dolomite of South Australia, finely laminated, micritic magnesite occurs rarely with mudcracks and tepee structures indicating subaerial exposure and evaporation, but more commonly as reworked, conglomeratic intraclasts (Frank & Fielding, 2003).

### 2.3. Effects of Deformation by Impact Shock

It is likely that areas of the ancient Martian magnesium carbonate system within the Jezero crater region have been affected by impacts. On Earth, we have observed impact structures in limestone and dolostone target rocks, but not magnesium carbonate rocks (e.g., Kieffer, 1971; Metzler et al., 1988; Pohl et al., 1977). Passage of the shock wave during impact events modifies the carbonate target rock to produce high-pressure deformation fabrics such as shatter cones and, where shock pressures are high enough, they can melt the carbonate target rock (e.g., Dence, 1971; Grieve et al., 1977). While devolatilization of carbonates was previously thought to be important, experiments and observations of terrestrial structures suggest that this process has minimal effect on Earth (Bell, 2016; Graup, 1999; Jones et al., 2005; Osinski & Spray, 2001). Instead, much of the carbonate is retained within carbonate-bearing impact melts (e.g., Graup, 1999; Jones et al., 2005; Osinski & Spray, 2001). These carbonate-bearing melts typically quench to form minerals, not glasses, and are immiscible with silicate impact melts (Freestone & Hamilton, 1980; Graup, 1999; Osinski & Spray, 2001). However, it is currently unknown how this melting process would work with the differing physical conditions of the Martian surface. Another open question is whether magnesite could form via quenching of magnesium carbonate impact melts. No such process has been observed on Earth, and the well studied Haughton structure showed carbonate melts were Mg-poor despite melting of dolomite within the target rock (Osinki & Spray, 2001; Osinski et al., 2005).

### 2.4. Magnesium Carbonates on Mars

Orbiter-based detection of magnesium carbonates is often associated with the detection of olivine-bearing rocks circumferential to the 1900-km Isidis impact basin, to the west in the Nili Fossae region and to the south in Libya Montes (Bishop et al., 2013; Bramble et al., 2017; Brown et al., 2020; Edwards & Ehlmann, 2015; Ehlmann et al., 2008; Mustard et al., 2009; Viviano et al., 2013) (Figure 1a). The olivine- and carbonate-bearing unit is typically a few to tens of meters thick and occurs in a distinctive stratigraphic position. It overlies an older, extensive low Ca-pyroxene and Fe/Mg-smectite-bearing rocks of Noachian age (>~3.8 Ga; Mandon et al., 2020; Scheller & Ehlmann, 2020) and underlies a high Ca-pyroxene-bearing mafic capping unit (Bramble et al., 2017). The olivine- and carbonate-bearing unit spans a large elevation range of more than 3 km. Formation hypotheses discussed in the literature include an olivine-bearing ashfall, lava flow, impact melt sheet, or igneous intrusion (Brown et al., 2020; Hamilton & Christensen, 2005; Hoefen et al., 2003; Kremer et al., 2019; Mustard et al., 2007, 2009; Tornabene et al., 2008).

The strong association between magnesium carbonates and ultramafic rocks on Earth suggests that potential ultramafic materials exposed in the watershed of the Jezero basin may host magnesium carbonates similar in context to the terrestrial examples discussed above. Complicating this interpretation, however, is the fact that modeling of mineralogy from orbital infrared datasets does not yield ultramafic compositions. Original thermal infrared modeling of olivine abundance estimated 30 wt. % olivine (Hoefen et al., 2003) and later analyses showed 10–15 wt. % olivine (Salvatore et al., 2018). Joint modeling of near-infrared and thermal infrared data suggests ~25 wt.% olivine, ~30 wt.% pyroxene, and ~15 wt.% carbonate (Edwards & Ehlmann, 2015). By contrast, unserpentinized ultramafic rocks on Earth are typically >80%–90% olivine and pyroxene. While it is possible that coverage by basaltic dust or sand obscures an ultramafic composition, it remains for in situ investigation to understand the composition of the rock and relationship between the olivine and carbonate. Observations of the carbonate textures, abundances of carbonate within the rocks, and whether they are vein-forming, nodule-forming, within the matrix of the olivine-bearing rock, or surficial (e.g., travertine, playa) will be critical for understanding the best terrestrial analogue environments for magnesium carbonate formation on Mars.

This laterally extensive, regional olivine-carbonate unit includes the watershed of Jezero crater within the extended mission area of Nili Planum. Similar materials are on the floor of Jezero crater and within its delta. The purest carbonate signatures regionally (i.e., lacking a Fe/Mg phyllosilicate absorption) are found in the sedimentary materials of Jezero crater (Ehlmann et al., 2008, 2009) (Figures 1b and 1c). The olivine-carbonate unit appears to drape over the Jezero crater rim to the floor, which would demand that it postdates formation of Jezero crater (Bramble et al., 2017; Brown et al., 2020; Goudge et al., 2015; Kremer et al., 2019; Mandon et al., 2020) (Figure 1). On the other hand, materials comprising the floor of Jezero could be distinctive deposits formed by sedimentation in a lake of the eroded olivine-carbonate formation. A separate magnesium carbonate-bearing unit has been hypothesized within the Jezero crater as the “margin light-toned fractured unit” in Stack et al. (2020) or “marginal carbonates” in Horgan et al. (2020) (Figure 1b). This unit has a stronger signal of magnesium carbonate than deltaic sediments or other floor units within beds of consistent elevational level with close proximity to the western part of the crater rim (Figure 1c). It is possible that this mode of magnesium carbonate accumulation represents authigenic precipitation from fluids that percolated down gradient in the shallow subsurface and then emerged at lake level to precipitate magnesium carbonate along the delta shoreline in a highstand position (Horgan et al., 2020). Alternatively and/or additionally, detrital carbonate grains transported from the regional olivine-carbonate unit outside Jezero crater were likely deposited fluvially in the prominent river delta in the crater (Goudge et al., 2015). Observations of the carbonate fabrics within the marginal carbonates of Jezero crater will likely be indicative as to whether these can be considered to be authigenic precipitants or detrital materials. Furthermore, observations of carbonate fabrics and structures can be tied to lake history and used to distinguish between an alkaline lake environment versus playas (Figure 2).

## 3. Magnesium Carbonate Fabrics and Paragenesis

**Figure 5.**
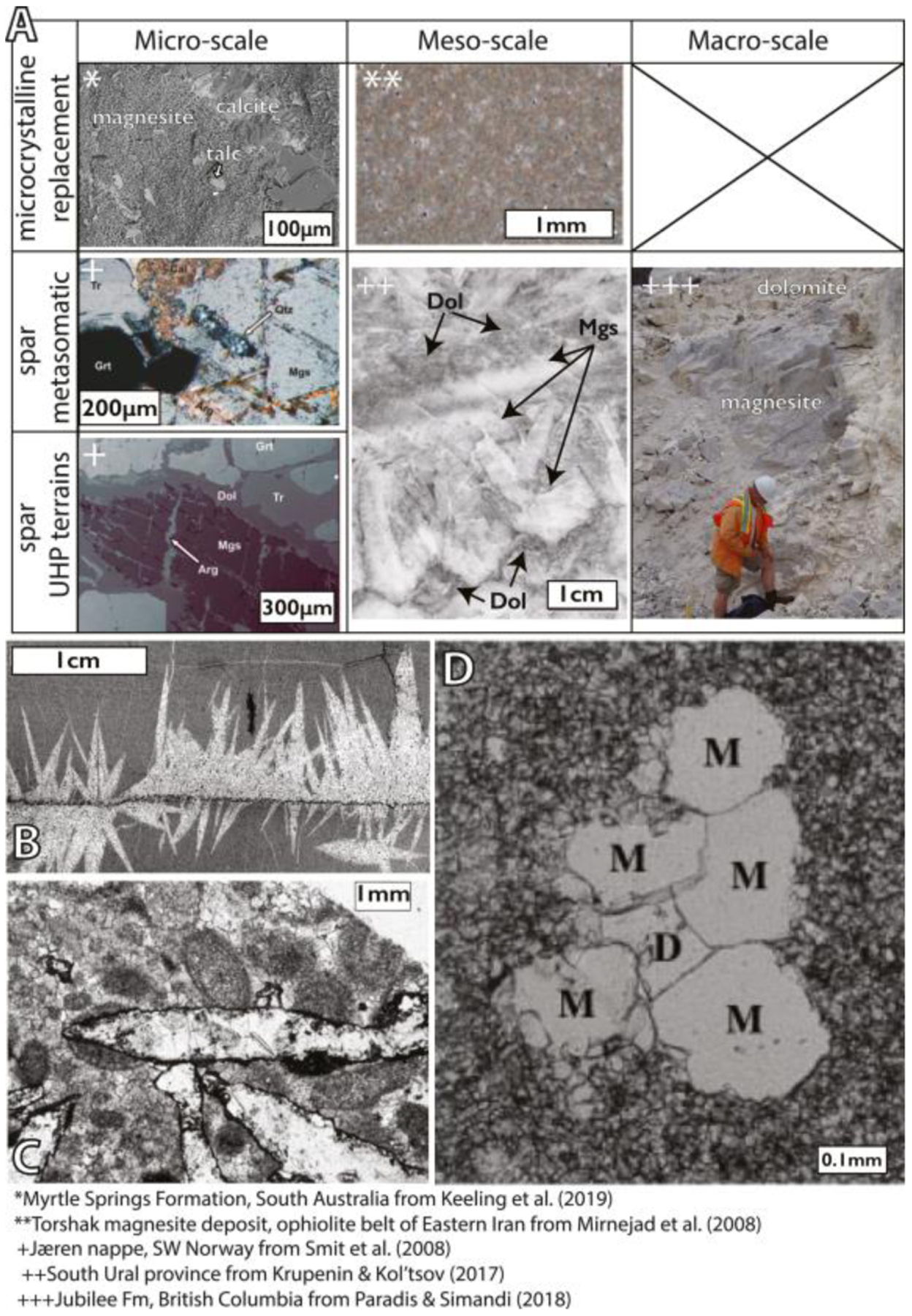
(a) Key textures at micro-, meso-, and macro-scale in diagenetic replacement environments (upper row) and metasomatic replacement as well as ultrahigh-pressure (UHP) environments (lower row). (b–d) Additional thin section micrographs show the diversity of magnesite replacement textures. (b) Lenticular sparry magnesite crystals growing from a stylolite and replacing dolomite in a metasomatic environment from Eugui-Asturreta magnesite deposit, Western Pyrenees, Spain (Lugli et al., 2000). (c) Lenticular sparry magnesite replacing oolitic dolomite rock from Burrano Fm, Northern Apennines, Italy (Lugli, Morteani & Blamart, 2002). (d) Neomorphic crystals of magnesite (M) and dolomite (d) and surrounding microcrystalline magnesite diagenetically replacing Paleoproterozoic dolostone from Tulomozerskaya Fm, Russian Karelia (Melezhik et al., 2001). The symbols denote the source of images from publications or contributed by individuals as listed in the bottom (Paradis & Simandl, 2018; Smit et al., 2008). Grt = garnet, Qtz = quartz, Mgs = magnesite, Dol = dolomite, Tr = tremolite, Arg = aragonite.

The occurrence, fabrics, paragenesis and associated mineral assemblages vary by environmental type, and environments of formation that can sometimes be inferred from these features (Table 1, Figures 3–5). The Perseverance rover is equipped with spectroscopic and imaging instruments that are specifically designed to determine rock textures and associated mineral assemblages. Hence, rover observations of fabrics and mineral assemblages can be used to directly test between environments of formation for magnesium carbonates on ancient Mars.

### 3.1. Ultramafic Rock-Hosted Magnesium Carbonate

In ultramafic terrains, magnesite occurs primarily within secondary vein deposits (Figure 3). Vein deposits from, for example, the Balkan Peninsula (referred to as Yugoslavia in past literature) occur at depths greater than ~80–200 m with a thickness of up to 20 m, then grading to more abundant, thin veins (1 mm–2 m wide) (Zachmann & Johannes, 1989). These veins are characteristically finely crystalline to microcrystalline of nm-μm scale crystals, often described as cryptocrystalline massive fabrics with occasional spheroidal textures (Figure 3) (Abu-Jaber & Kimberley, 1992). The spheroidal fabric is characterized by ~3 mm aggregates of fine euhedral crystals (<1 mm) within a matrix of finer (<0.1 mm) spherical grains. At the macroscopic scale, microcrystalline magnesite is bright white. Microscopically, it exhibits a characteristic conchoidal fracture, and is devoid of fluid inclusions or pseudomorphic textures after the parental ultramafic rock (García del Real et al., 2016). The massive microcrystalline magnesite forms vein fills together with other Ca-carbonates, such as dolomite and calcite, as well as silica and serpentine within serpentinized dunite or peridotite (Abu-Jaber & Kimberley, 1992; Zachmann & Johannes, 1989) (Figure 3). In addition, ultramafic rocks may host multiple episodes of vein formation in which some veins are primarily composed of calcite whereas others are primarily composed of magnesium carbonates (Cooperdock et al., 2020; Streit et al., 2012).

In carbonated ultramafic rocks, magnesium carbonates form assemblages with other Fe- and Ca-carbonates, silica, sulfides, spinel, iron oxides, fuchsite, serpentine, and talc (Boskabadi et al., 2020) and typically occur in close association with serpentinized dunite or peridotite in the (Section 2). In carbonated ultramafic rocks, magnesium carbonate occur both as veins and within the matrix forming finely crystalline to microcrystalline or cryptocrystalline textures with nm-μm scale crystals finely intergrown with silica and other minor phases (Boskabadi et al., 2020; Falk, 2014). However, coarse magnesite crystals of 1–0.25 mm can be found in alternating layers in carbonated ultramafic rocks as well (Menzel et al., 2018). Importantly, the specific mineral assemblages found within the carbonated ultramafic rock can be used to constrain the temperature and pressure conditions of formation.

### 3.2. Sedimentary Deposits

#### 3.2.1. Observed Fabrics and Parageneses

In Late Triassic hypersaline evaporite deposits (Lugli et al., 2002) and in Holocene salt lake deposits (Mees & Keppens, 2013), magnesite and nesquehonite seem to have precipitated together with evaporitic sulfate salts and halite (Figure 4). Sedimentary magnesite deposits also occur in the Precambrian rock record (Alderman & von der Borch, 1961; Melezhik et al., 2001). Here, Ca-bearing sulfate minerals are likely absent due to both the depleted marine sulfate reservoir (compared to present day), and the seawater chemistry that favored Ca-carbonate precipitation instead of Ca-bearing sulfate (Frank & Fielding, 2003; Grotzinger, 1989; Grotzinger & Kasting, 1993).

Magnesite, hydromagnesite, dypingite, and nesquehonite assemblages have been found in playas within Atlin, British Columbia, along with aragonite, quartz, clay minerals, and feldspars (Power et al., 2007). These form large, white mounds with cauliflower textures (Figure 4) (Power et al., 2019). It is thought that the formation of dypingite serves as a precursor for the formation of hydromagnesite via dehydration at the playa mounds in Atlin (Power et al., 2007). Similarly, beds of magnesite, hydromagnesite, dolomite, aragonite, and gypsum are found in a series of alkaline lakes associated with the Coorong Lagoon, east coast of South Australia (von Der Bock, 1965; Warren, 1990). These carbonate assemblages form laminated or massive lakebeds and nodules in siliciclastics (Rosen et al., 1988; Warren, 1990). The relative influence of marine, fluvial and groundwaters input and evaporation controlled the specific mineralogical assemblages. Mg input results from groundwaters interacting with terrestrial materials including the local Pleistocene dune system, and the relative importance of (hydro)magnesite decreases with marine influence and increases with evaporation and salinity. As Mg-enriched groundwaters feed the northern Coorong lakes, Ca-rich phases precipitate closer to inputs at lake margins, and Mg-rich phases precipitate basinward where evaporation dominates (Warren, 1990).

#### 3.2.2. Preservation of Microbial Mats

Microbial mats can be associated with hydrated magnesium carbonates in both modern and ancient environments, mostly in alkaline lakes but occasionally in hypersaline lakes as well (Figure 4) (e.g., Braithwaite & Zedef, 1994; Cabestrero & Sanz-Montero, 2018; Power et al., 2019; Renaut, 1993; Sanz-Montero & Rodríguez-Aranda, 2012; Walter et al., 1973). Microbial-mat associated magnesium carbonates form laminated, clotted, and spherical textures that make up stromatolitic or thrombolitic structures (Bosak et al., 2021) (Figure 4). The Perseverance rover will be able to analyze for the presence of these macroscopic and microscopic microbial mat associated textures and structures (Bosak et al., 2021). Although no single textural observation can be utilized to determine unequivocally that biosignatures are recorded within the magnesium carbonate deposit, the presence of these textures are indicative that sampling and subsequent laboratory measurements to understand the formation of these structures would be highly informative.

Laminated fabrics of primarily hydromagnesite makes up thrombolites and stromatolites in well studied modern alkaline lake environments within the Las Eras lake, Spain (Sanz-Montero et al., 2019), Lake Salda, Turkey (Braithwaite and Zedef, 1994), and Lake Alchichica, Mexico (Kaźmierczak et al., 2011). In Las Eras lake, hydromagnesite, magnesite, and dolomite thrombolites are associated with cyanobacterial mats enriched in extrapolymeric substances (EPS), sometimes in association with sulfate salts (Sanz-Montero et al., 2019). Hydromagnesite aggregates nucleate EPS-encased microorganisms, in association with nesquehonite crystals to form coatings (Sanz-Montero et al., 2019). Dolomite and magnesite also form aggregates of nanoparticles that rest on the hydromagnesite-coated EPS (Sanz-Montero et al., 2019). In Lake Salda, stromatolites form in sizes ranging from a few cm in height in shallow water to 1–2 m at depths of 6–8 m below present lake level (Figure 4) (Braithwaite & Zedef, 1996). The stromatolites contain laminae up to a few centimeters thick with distinct regions of ~5 μm spherical or bladed hydromagnesite-coated filaments and diatoms (Figure 4) (Braithwaite & Zedef, 1996). Mg-silicates have been reported in modern Lake Salda stromatolites both as authigenic minerals and as trapped detrital grains (Balci et al., 2020), which is a common mineralogical component in highly alkaline systems (e.g., Darragi & Tardy, 1987). Lake Salda stromatolites are dominated by cyanobacteria and gamma and alpha-proteobacteria (Balci et al., 2020). In Lake Alchichica, Mexico, stromatolites, massive domes, and crusts are covered by a cyanobacteria-hosted biofilm that contains trapped hydromagnesite and aragonite inside an EPS matrix (Gérard et al., 2013; Kaźmierczak et al., 2011). In these systems, research is still needed in order to understand whether magnesium carbonates are precipitated authigenically within biofilms or if biofilms trap and bind the magnesium carbonates (see Section 4.3).

Microbial mat associated textures can be retained within ancient magnesium carbonate deposits, like those expected on Mars. However, magnesium carbonates, especially magnesite, may not be authigenic and could represent instead diagenetic recrystallization of Ca-carbonates or hydrated magnesium carbonates. Magnesite in ancient Paleoproterozoic basin deposits in the Liaodong Peninsula, China, forms stromatolitic structures as well as thinly laminated beds that are preserved in, for example, the Dashinqiao Fm, interpreted to have formed in shallow marine settings (Dong et al., 2016; Zhang, 1988). These stromatolites and laminated beds reveal complex associations between primary sedimentary magnesite with dark, microcrystalline microtextures and later coarse, sparry diagenetic magnesite replacement fabrics (Dong et al., 2016; Zhang, 1988). Similarly, in the Paleoproterozoic Tulomozerkskaya Fm, Russia, magnesite is present in stromatolitic structures and laminated beds but are not interpreted to be primary precipitants and represent instead early diagenetic replacements of associated dolomite (see section 3.3).

### 3.3. Diagenetic, Metasomatic, and Metamorphic Replacement

Sedimentary deposits, or *strata-bound* deposits, of limestone and dolostone formed in lacustrine and marine environments can be replaced by later magnesite when exposed to diagenetic or burial fluids that have migrated through ultramafic terrain (Figure 5). Magnesite replacement can either preserve the original bedding and associated textures or form massive, fabric-destructive microcrystalline textures (Figure 5) (Abu-Jaber & Kimberley, 1992).

Microcrystalline replacement magnesite with occasional neomorphic coarse crystals represents synsedimentary or early diagenetic replacement and forms with diagenetic sulfates and quartz (Abu-Jaber & Kimberley, 1992; Keeling et al., 2019; Melezhik et al., 2001) (Figures 5a and 5d). Under relatively low-pressure burial conditions with temperatures at 100°C–400°C, metasomatism of primary dolostone by hydrothermal Mg-rich fluids leads to the formation of sparry magnesite (Figures 5a–5c) (Aharon, 1988; Lugli, Morteani & Blamart, 2002). This metasomatic magnesite spar is characterized by a medium (1–9 mm) to coarse (1–15 cm) crystal size, and can occur as massive deposits (Figure 5a), fracture fillings, or with porphyroblastic (scattered lens-shaped crystals in dolostones and sulfate rocks) (Figures 5b–5c), rosette, banded, and palisade (Figure 5d) textures (Lugli et al., 2002; Ronchi et al., 2008). At higher temperatures and pressures, metasedimentary, sparry magnesite has been found in association with tremolite-quartz-calcite ± talc ± diopside (Krupenin & Kol’tsov, 2017; Ronchi et al., 2008). In ultrahigh-pressure (UHP) metamorphic terrains, magnesite occurs as sub-mm grains with diopside in assemblages of garnet websterites, peridotites and eclogites (Figure 5) (Zhang & Liou, 1994; Yang et al., 1993).

## 4. Thermodynamics and Kinetics of Magnesium Carbonate Precipitation

As with other carbonates, thermodynamic and kinetic factors regulate precipitation of magnesium carbonates. Direct precipitation of terrestrial magnesite is not fully understood but can be favored by high temperatures, dehydration of preexisting hydrated magnesium carbonates, or perhaps microbially mediated processes. In a Martian context, it will therefore be important to make careful observations of pseudomorphic textures, small-scale chemistry and crystal structure, or other evidence to differentiate processes.

### 4.1. Thermodynamics

Magnesite forms in aqueous solution by the addition of magnesium and carbonate ions (Equation 1). Equilibrium between magnesite and its constituent ions in solution is dictated by the solubility constant, *K*_sp_, for a given temperature and pressure. *K*_sp_ is defined by the product of the activity of the magnesium $\left(a_{{\text{Mg}}^{2+}}\right)$ and carbonate $\left(a_{{\text{CO}}_{3}^{2-}}\right)$ ions in solution at equilibrium, divided by the activity of solid magnesite $\left(a_{{\text{MgCO}}_{3}}\right)$ As pure solids are generally assumed to have an activity of unity, *K*_sp_ may be simplified to the product of the magnesium and carbonate ion activities (Equation 2). Experimental determination of *K*_sp_ at 25°C is complicated by long equilibration times between magnesite and solution at low temperatures. As a result, determination of *K*_sp_ values at ambient temperature rely on extrapolation of high temperature experimental data where magnesite precipitates readily. Reported *K*_sp_ values and other thermodynamic constraints on the precipitation and dissolution of magnesite vary (Allison et al., 1991; Bär, 1932; Christ & Hosteltler, 1970; Halla & Van Tassel, 1966; Langmuir, 1965; Sayles & Fyfe, 1973); generally accepted *K*_sp_ values are between 10^−8.2^ and 10^−7.8^ (Bénézeth et al., 2011; Robie & Waldbaum, 1968; Rossini, 1961).

(1) $${\text{MgCO}}_{3}\left(s\right)⇌{\text{Mg}}^{2+}+{\text{CO}}_{3}^{2-}$$

(2) $$K_{\text{sp}}^{\text{mgs}}=\frac{a_{{\text{Mg}}^{2+}}\times a_{{\text{CO}}_{3}^{2-}}}{a_{{\text{MgCO}}_{3}}}≅a_{{\text{Mg}}^{2+}\left(eq\right)}\times a_{{\text{CO}}_{3}^{2-}\left(\text{eq}\right)}$$

The thermodynamic driving force for magnesite precipitation from aqueous solution can be defined by the saturation state (Ω), the ratio between the ion activity product (IAP) of reactive species and the solubility product (Equation 3).

(3) $$\Omega =\frac{\text{IAP}}{K_{\text{sp}}}=\frac{a_{{\text{Mg}}^{2+}}\times a_{{\text{CO}}_{3}^{2-}}}{K_{\text{sp}}^{\text{mgs}}}$$

Precipitation is favored when Ω > 1 and dissolution is favored when Ω < 1. Magnesite is more soluble than other naturally occurring metal-carbonate phases: $K_{\text{sp}}^{\text{magnesite}}>K_{\text{sp}}^{\text{calcite}}>K_{\text{sp}}^{\text{strontanite}}>K_{\text{sp}}^{\text{siderite}}>K_{\text{sp}}^{\text{rhodochrosite}}>K_{\text{sp}}^{\text{dolomite}}$ (Stumm & Morgan, 1996). Even when conditions of supersaturation are reached (Ω > 1), magnesite is rarely observed to nucleate and form as a primary precipitate in aqueous solution under ambient conditions, indicating that kinetic factors likely prevent direct magnesite precipitation from solution. Direct precipitation from supersaturated solutions in experiments has only been observed above 100°C (Hänchen et al., 2008), or at lower temperatures when aided by microbial materials and metabolism (see Section 4.3).

Magnesite formation at ambient temperatures and atmospheric pressures has been observed via recrystallization of hydrated magnesium carbonates (Deelman, 1999; Giammar et al., 2005; Hänchen et al., 2008; Hopkinson et al., 2012; Königsberger et al., 1999). The relative thermodynamic stabilities of the common phases in this system are magnesite > hydromagnesite > nesquehonite, above 8.5°C, or lansfordite, below 8.5°C (Marion, 2001). Despite the inferior energetics, the hydrated phases appear to be less inhibited kinetically, may therefore precipitate before magnesite and then recrystallize to form the more thermodynamically stable magnesite. In Jezero crater and its watershed, infrared spectroscopic data are compatible with hydrous carbonates as a component with magnesite. Hence, we may find either magnesite, hydromagnesite, or a combination of these and other carbonate minerals within the landing site (see Section 1).

### 4.2. Kinetics

Kinetic factors also impact magnesite precipitation and dissolution rates. Like calculation of the *K*_sp_, and kinetic data are experimentally determined at elevated temperatures and pressures and extrapolated to ambient conditions (Saldi et al., 2009; Sayles & Fyfe, 1973). Alternatively, kinetic data are obtained for the transformation of metastable phases to magnesite and can more aptly be defined as a “recrystallization” rate (Zhang et al., 2000). For solutions at Ω = 10 and 25°C, the rate of magnesite precipitation was calculated to be on the order of 10^−18^ mol cm^−2^ s^−1^, a rate that is six orders of magnitude slower than that of calcite formation at 25°C and similar oversaturation (Saldi et al., 2009). Power et al. (2019) calculated a slightly higher magnesite precipitation rate of 10^−17^–10^−16^ mol cm^−2^ s^−1^ within playas in Atlin, British Columbia.

Sayles and Fyfe (1973) observed an increase in the rate of magnesite precipitation with increasing pCO_2_ and ionic strength and decreasing magnesium ion concentration (in contrast to thermodynamic predictions). Other phases in the MgO-CO_2_-H_2_O system do not experience kinetic sluggishness to the same extent as magnesite, and thus can facilitate magnesite formation (via recrystallization of hydrated precursors) on the order of hours (Felmy et al., 2012; Sayles & Fyfe, 1973; Schaef et al., 2011; Zhang et al., 2000).

The slow precipitation kinetics of magnesite are thought to originate from effects between the magnesite surface and magnesium ions. In most environmental waters, magnesium ions are strongly complexed with 6–12 water molecules that exchange slowly (Di Tommaso & de Leeuw, 2010; Jiao et al., 2006). The slow desolvation of magnesium ions at the magnesite surface effectively retards magnesium incorporation and attachment of carbonate ions onto the crystal surface (Lippmann, 1973; Saldi et al., 2009). The relationship between magnesium ions and kinetic inhibition of mineral growth has been extensively studied in calcite (e.g., Bischoff, 1968). Solvated magnesium ions inhibit calcite growth by acting as “kink blockers” (Davis et al., 2000; Mavromatis et al., 2013; Wasylenki et al., 2005). Magnesium ions will adsorb to high energy surface sites (kinks) with waters of hydration still complexed. This effectively blocks the attachment of additional monomers to the surface until the complex dehydrates and incorporates into the crystal or the complex desorbs and returns to solution (Davis et al., 2000; Mavromatis et al., 2013; Wasylenki et al., 2005). At higher temperatures, magnesium ions exchange H_2_O more rapidly, resulting in the observed trend of quicker rates at higher temperatures (Di Tommaso & de Leeuw, 2010). This concept was confirmed experimentally by Xu et al. (2013), although there may be an unknown additional inhibiting barrier to magnesium carbonate growth that is still the subject of study.

### 4.3. Studies of Microbial Influences on Precipitation

Laboratory experiments have demonstrated the ability of microorganisms to influence precipitation of magnesium carbonates. Biotic process is a viable way to overcome the thermodynamic and kinetic barriers outlined above. However, further research is still needed in order to fully confirm these biotic models for magnesium carbonate precipitation. Cultures of cyanobacteria isolated from Fayetteville Green Lake, New York (Thompson & Ferris, 1990), playas within Atlin, British Columbia (Power et al., 2007), and Lake Salda (Shirokova et al., 2013) were found to promote magnesium carbonate precipitation through either increasing pH or providing cell surfaces that can induce the dehydration, concentration, and/or binding of Mg^2+^ ions (Moore et al., 2020; Renaut, 1993). However, precipitation rates of hydrous magnesium carbonates were not found to be affected by the presence of cyanobacteria as compared with an abiotic control within Mavromatis et al. (2012).

In other studies, EPS has been found to play a large role in magnesium carbonate precipitation. In comparisons between laboratory experiments and Las Eras microbial mats, hydromagnesites were proposed to form early on by nucleating on EPS, while anhydrous magnesite and dolomite formed at later mat decay stages by nucleating on bacterial nanoglobules and/or collapsed cells (Sanz-Montero et al., 2019). The study proposed that as organic substrate declined, heterotrophs such as Firmicutes reduced metabolic activity and started to produce nanoglobules, causing a change in available substrates and the nucleation of dolomite and magnesite instead of hydrous magnesium carbonates (Sanz-Montero et al., 2019). Hydromagnesite within Lake Salda microbial mats were similarly proposed to nucleate on degraded EPS caused by heterotrophic nanoglobule formation (Balci et al., 2020). Alternatively, the degradation of EPS itself has been proposed to release Mg^2+^, thereby directly promoting saturation of magnesium carbonates (Kaźmierczak et al., 2011).

## 5. Isotopic and Elemental Constraints on Magnesium Carbonate Formation

Stable and clumped isotope data derived from magnesite and/or hydrated magnesium carbonates can be used to place constraints on mechanisms of their formation and past environmental conditions. The carbon isotopic composition may record the composition of the magnesite carbon source, and δ^18^O and Δ_47_ may provide constraints on the composition and temperature of magnesite-forming waters, both of which provide critical information related to the setting and processes of magnesite formation. For Mars, knowledge of the carbon and oxygen stable isotopic evolution of its atmosphere is limited to analysis of meteorites and measurements by the Mars Science Laboratory (MSL) rover, Phoenix lander, and Viking landers (Carr et al., 1985; Nier & McElroy, 1977; Niles et al., 2010; Webster et al., 2013; Wright et al., 1992). Assuming that organic matter was not a dominant carbon source on Mars, the δ^13^C of Martian magnesites from Jezero crater may provide insight into atmospheric CO_2_ values from ancient Mars and constrain the evolution of the Martian atmosphere (Franz et al., 2020; Hu et al., 2015). This assumption is supported by a study of siderite-magnesite carbonate globules in the Martian meteorite ALH84001, indicating that these formed at low temperature from subsurface water with CO_2_ derived from the Martian atmosphere (Halevy et al., 2011). Therefore, δ^18^O measurements of Jezero magnesium carbonates may further contextualize the evolution of the oxygen isotopic budget throughout Mars’ history and potentially identify the aqueous environment of magnesite formation in Jezero crater (Heard & Kite, 2020; Jakosky et al., 2018).

Carbon sources, fluid sources, and temperature of formation as well as diagenetic overprints have been identified using isotopic and elemental chemistry techniques on terrestrial samples and would be similarly applicable to magnesium carbonate samples returned from Mars. At the same time, continued studies on the application and interpretation of these techniques on terrestrial magnesium carbonates will help us better understand magnesium carbonate formation conditions in general and help us interpret these chemical signals within returned Martian samples.

### 5.1. Carbon Sources

There is evidence that the processes involved in fractionating δ^13^C within carbon in the precipitating system in terrestrial sedimentary and ultramafic systems differ, and thus can be used to distinguish different environmental conditions of magnesite formation. On Mars, we expect carbon to be primarily sourced from the atmosphere and mantle with minor contributions from meteoritic and potentially endogenous organic matter. The δ^13^C of carbonates can be used to track changes in atmospheric composition and input of volcanic gases. Detailed analyses of δ^13^C can be used to identify other potential sources of carbon, mixing of these endmembers, and track processes such as evaporation. These are particularly important measurements to be made not only for interpreting carbonate formation processes, but also for understanding why the CO_2_-dominant atmosphere on Mars did not form abundant carbonate on the Martian surface (e.g., Franz et al., 2020; Hu et al., 2015).

**Figure 6.**
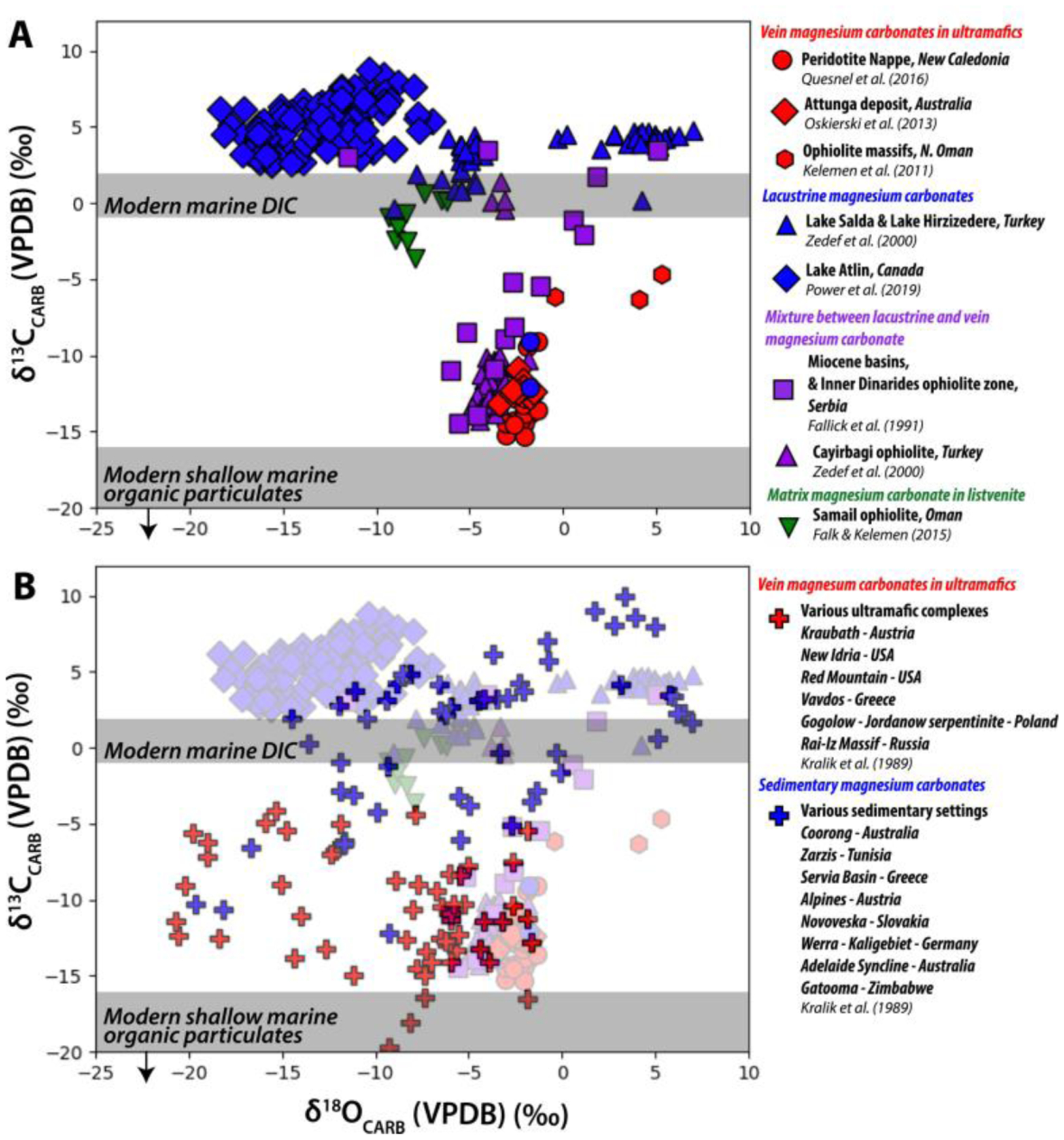
(a) Recently published isotopic compositions of major magnesium carbonate deposits from veins in ultramafic complexes (red symbols) and lacustrine settings (blue symbols). Vein and lacustrine magnesium carbonates occupy two different endmember fields within the δ^13^C-δ^18^O space. Certain samples reflect mixing between endmembers, e.g., mixing between vein and lacustrine magnesium carbonate or mixing between an organic and atmospheric carbon source (purple squares). The large δ^18^O variation between sedimentary systems is driven by the local lake water composition as related to precipitation patterns, while δ^18^O variation within a single lake system is attributed to evaporation effects enriching δ^18^O. Green triangles show the isotopic composition of matrix magnesites within listvenite from the Semail ophiolite, Oman (Falk & Kelemen, 2015). The δ^13^C composition of vein and listvenite magnesite from Oman are higher compared with other vein magnesite and are thought to be derived from local calcite-bearing metasediments. (b) Comparison of data from panel A with a compilation by Kralik et al. (1989) of isotopic compositions of major deposits of vein (red plusses) and sedimentary (blue plusses) magnesium carbonate from around the world. Note that vein and sedimentary magnesium carbonate still appear to occupy separate fields in the δ^13^C-δ^18^O space, but the boundaries between endmembers are no longer clear-cut. Example, ranges of modern marine DIC (Kroopnick, 1985) and the upper range of modern shallow marine organic particulates (Freeman, 2001) are shown as gray bars for comparison with data. Arrow shown to note that δ^13^C of organic particulates can reach down to −36‰ but full range is not shown here.

Magnesite veins in ultramafic complexes record δ^13^C values (−20 to −4‰ VPDB) lower than carbonates that precipitate from the typical range of Earth surface dissolved inorganic carbon (DIC) δ^13^C values (Figure 6) (Kralik et al., 1989; Schroll, 2002). Low δ^13^C values have been interpreted as the result of either decarboxylation of organic matter within ocean sediments in hydrothermal systems or incorporation of inorganic carbon derived from organic matter remineralization in the soils and regolith through meteoric infiltration (Kelemen et al., 2011; Quesnel et al., 2013, 2016; Schroll, 2002; Zedef et al., 2000). The higher vein magnesite δ^13^C values typically are interpreted to record mixing with an atmospheric carbon component as values are closer to those expected for carbonates precipitated from DIC (Oskierski et al., 2013; Quesnel et al., 2013, 2016; Schroll, 2002; Zedef et al., 2000). For veins and listvenites specifically in the Semail Ophiolite, Oman, the source of carbon may be local sediments as δ^13^C is similar to that of local calcite-bearing metasediments (Falk & Kelemen, 2015; Kelemen et al., 2011).

Magnesite that forms in soil and sedimentary environments records higher δ^13^C values of 0–10‰ VPDB (Figure 6) representing atmospheric CO_2_ (Fallick et al., 1991; Kralik et al., 1989; Melezhik et al., 2001; Power et al., 2019; Schroll, 2002; Zedef et al., 2000). Magnesite with enriched ^13^C (>0‰) has been interpreted to record the influence of evaporation and CO_2_ degassing during precipitation (Figure 6) (Melezhik et al., 2001; Power et al., 2014, 2019). Playa and lacustrine magnesite deposits can record fluctuations in lake levels and temperatures driven by climate change (via temperature and hydrology) in their carbon isotope compositions (Andrews, 2006; Li & Ku, 1997). Such measurements of magnesium carbonates from within the Jezero lake system may provide valuable constraints on any climatic fluctuations on ancient Mars that controlled the isotopic composition of the lake water.

### 5.2. Fluid Sources

Detailed measurements of oxygen isotopes within Martian magnesium carbonates are important because they may allow us to determine the fluid sources that precipitated carbonates on Mars. The oxygen isotopic composition of carbonate (δ^18^O) is a function of both the δ^18^O of the fluid source from which the carbonate precipitated and the temperature of carbonate formation (Epstein et al., 1953; McCrea, 1950). Water δ^18^O can be directly calculated if the temperature can be independently assessed, for example, by using clumped isotope temperature (T(Δ_47_)) (e.g., Kim & O’Neil, 1997). Currently, hypotheses for Martian carbonate formation include hydrothermal alteration processes, weathering processes, or precipitation within a sedimentary system (see Sections 1 and 2). Determining the δ^18^O composition of carbonates will allow us to directly test between these three different fluid sources. At the same time, we must also work toward a better understanding of these isotopic systems on Earth in order to apply robust interpretations to the Martian returned samples.

Very low magnesite δ^18^O compositions (−20–0‰ VPDB; Figure 6) in combination with T(Δ_47_) values approximating plausible Earth surface temperatures (20°C–60°C, Kelemen et al., 2011; Quesnel et al., 2016) are interpreted to record magnesite formation from meteoric fluids that exchanged with ultramafic rock during downward infiltration (Kelemen et al., 2011; Oskierski et al., 2013; Quesnel et al., 2013, 2016). Alternatively, some interpretations of δ^18^O and clumped isotope values from magnesite in ultramafic complexes have led researchers to believe that magnesite formed hydrothermally and/or during burial at 70°C–100°C (Falk & Kelemen, 2015; Fallick et al., 1991). In summary, better understanding of the source fluids that form ultramafic rock-hosted magnesium carbonates is an ongoing pursuit and the sources of oxygen in the magnesium carbonates in ultramafic veins remain somewhat mysterious requiring further study (Falk et al., 2016; Kelemen et al., 2011; Oskierski et al., 2013; Quesnel et al., 2013, 2016; Zedef et al., 2000).

By contrast, sedimentary magnesite typically clearly records the water composition of their depositional environments (Figure 6) (Fallick et al., 1991; Kralik et al., 1989; Power et al., 2014, 2019; Zedef et al., 2000). In evaporative lacustrine settings, magnesite may retain both low δ^18^O reflective of meteoric fluids and high δ^18^O resulting from progressive evaporation within samples from a single lake system (Figure 6) (Power et al., 2019; Zedef et al., 2000). Similar to δ^13^C, the oxygen isotopic composition of sedimentary carbonates can record climatic fluctuations that control lake water composition (Andrews, 2006; Li & Ku, 1997). Combined δ^13^C and δ^18^O data of systems that include both vein and lacustrine magnesite reveal an isotopic mixing line with two endmembers: (a) a lacustrine, low-T endmember with variable δ^18^O due to evaporative effects and high δ^13^C where carbon was sourced from atmosphere, and (b) a hydrothermal endmember and low δ^13^C environment where carbon was sourced from oxidation of organic matter (Figure 6) (Fallick et al., 1991; Zedef et al., 2000).

Finally, it is important to consider possible diagenetic overprinting of isotopic compositions, which is common in many carbonates. Magnesium carbonates are highly soluble and thus prone to dissolution and recrystallization. In addition, primary magnesite and hydromagnesite isotopic signatures may be changed during dehydration of more hydrated magnesium carbonate phases (Power et al., 2019). Understanding whether or not a given sample may have experienced such post-deposition diagenetic overprints of isotopic compositions is particularly important to Mars, as colder surface temperatures on Earth favor the formation of hydrated magnesium carbonates as precursors to magnesite (see Section 4). Studies of dehydration processes of hydrated Ca-carbonate conversion to calcite/aragonite show that δ^18^O can re-equilibrate due to exchange between CO_3_- and H_2_O-associated oxygen as water is lost during heating (Scheller et al., 2020). As such, it is critical to understand the isotopic effects of dehydration and diagenesis in Martian samples in order to make inferences about the carbon and fluid source of the precursor mineral.

### 5.3. Mg Sources

The Mg-isotope systematics of magnesite have the potential to trace Mg sources and aqueous processes such as weathering and carbonation, that leads to magnesite formation on Earth and by extension on Mars. Mg has three stable isotopes of ^24^Mg, ^25^Mg, and ^26^Mg. In general, magnesite minerals are depleted in ^26^Mg compared with their source fluid, with the offset determined by a temperature-dependent fractionation factor (de Obeso et al., 2020; Oskierski et al., 2019; Pearce et al., 2012). As such, the Mg isotope composition of carbonates can be used to trace the fluid/rock source of Mg (Tipper et al., 2006). However, this interpretive framework is complicated by Mg isotope fractionations related to aqueous reactions (Blättler et al., 2015). Magnesite in ultramafic rocks appears to be ^24^Mg-enriched by a few permil compared with their expected equilibrium value (Oskierski et al., 2019), which may result from carbonation reactions (Beinlich et al., 2014). Oskierski et al. (2019) demonstrated that nodular magnesite, formed through chemical weathering, may record a greater enrichment in ^24^Mg compared with vein magnesite in the same system due to interspecies Mg isotope fractionations during dissolution/reprecipitation. While it has been proposed that Mg isotopes may record biological fractionations, initial natural and experimental magnesite formed in biotic and abiotic conditions appear to record the same Mg isotope fractionation (Mavromatis et al., 2012; Shirokova et al., 2013).

### 5.4. Temperature of Precipitation and Tracking Disequilibrium Conditions

In cases, where fluid inclusions are preserved, fluid inclusion thermometry can be utilized to measure the temperature of the captured fluid. Fluid inclusion thermometry has been successfully applied to magnesium carbonates in listvenites and veins within ultramafic rock-hosted deposits, yielding 210°C–250°C (Hansen et al., 2005; Schandl & Wicks, 1993). Alternatively, carbonate clumped isotope thermometry is a technique used to determine the temperature of formation of carbonate minerals by measuring the abundance of the mass-47 isotopologue (Δ_47_) of CO_2_ (^13^C^18^O^16^O, “clumped species”) released after digestion of a carbonate mineral. Clumped isotope thermometry also allows for the calculation of oxygen isotopic composition of the precipitating fluid (e.g., Daeron et al., 2011; Eiler, 2011, 2007; Fernandez et al., 2014; Huntington & Lechler, 2015; Huntington et al., 2011; Lloyd et al., 2017; Ryb et al., 2017). The basis for this measurement is the fact that the abundance of ^13^C–^18^O bonds within the solid is temperature dependent (Ghosh et al., 2006). Therefore, clumped isotope thermometry is a highly valuable technique applicable to Martian returned samples that can aid in determining fluid sources and carbonate formation environments on ancient Mars (see Section 5.2). However, this thermometer is well established for calcite and dolomite, but is currently being developed for siderite and magnesite (e.g., Kelemen et al., 2011; van Dijk et al., 2019).

Initial clumped isotope studies have been used to interpret conditions of magnesite genesis and the isotopic composition of the precipitating fluid (e.g., García del Real et al., 2016; Kelemen et al., 2011; Quesnel et al., 2016). Most ultramafic rock-hosted vein magnesite generally yield low temperatures of 15°C–50°C from clumped isotope thermometry indicative of formation through meteoric fluids (García del Real et al., 2016; Kelemen et al., 2011; Quesnel et al., 2016; Streit et al., 2012). Falk and Kelemen (2015) found higher clumped isotope temperatures (37°C–114°C) of dolomite and magnesite formed through carbonation processes. Quartz-magnesite and talc-magnesite mineral pair oxygen isotope measurements were instead used to constrain vein and listvenite formation temperatures of 249°C–287°C within the Linnajavri ultramafic complex, Norway (Beinlich et al., 2012). Lake magnesite yields low clumped isotope temperatures of 6°C–14°C for Altin Playa, Canada (Power et al., 2019). Last, clumped isotopes of coarse crystals of magnesite within metamorphic rocks can record very high temperatures of 300°C–650°C (García del Real et al., 2016).

The present state of the literature reveals some discrepancies between calculated clumped isotopes temperatures and other data, indicating that more work is needed for reliable magnesium carbonate paleothermometry. There are a number of methodological challenges with regard to measuring and analyzing magnesite clumped isotopes that require further study, including (a) ensuring complete liberation of CO_2_ from magnesite during acid digestion, (b) determining the correct acid digestion fraction factor for magnesite, (c) determining the appropriate calibration curve for deriving temperatures from clumped isotope values, and (d) determining the temperature dependent magnesite-fluid and magnesite-CO_2_ fractionation factors for oxygen and carbon isotopes. Furthermore, clumped isotope thermometry is only applicable when carbonates formed under equilibrium conditions. Disequilibrium-degassing, thermal perturbation, kinetic effects, shock effects, biological effects, and later heating events above the equilibrium blocking temperature (~145°C–235°C for calcites, Lloyd et al., 2018; Stolper & Eiler, 2015) can also affect the clumped isotopic signatures and require further study. In fact, the second important use of measuring Δ_47_ is to identify carbonate formation under disequilibrium conditions. Ca-carbonate formation at disequilibrium has been confirmed using clumped isotopes (e.g., Falk et al., 2016). In these cases, disequilibrium and kinetic effects can result in large ranges of Δ_47_ and associated δ^13^C and δ^18^O that otherwise would have been falsely interpreted as a primary signal (e.g., Falk et al., 2016). Hence, application of clumped isotope techniques on returned Martian samples would be highly important in order to interpret both the temperature of carbonate formation and the origin of δ^13^C and δ^18^O isotopic compositions.

#### 5.4.1. Elemental Constraints

Major, minor, and trace element concentrations in magnesite are diagnostic tools that have been used to detect detrital contamination, source rocks influence, diagenetic overprints, and redox conditions. Magnesites found in ultramafic environments typically have a very restricted range of trace elements (i.e., those that substitute for Mg mostly). Kuşcu et al. (2017) measured trace element concentrations in vein magnesite from the Madenli area of the Sarkikaraagac Ophiolite, sedimentary hunite-magnesite deposits in Miocene-Pliocene lacustrine rocks in the Asagitirtar area, and hydromagnesite near Lake Salda in the lacustrine basin on the Yesilova ophiolites. They attributed that variations in trace element content between the sites was likely due to fluid percolation through local volcanic basement rock rather than processes related to variability in temperature or pressure. Lugli et al. (2000) observed positive correlations between trace elements and Al and K and attributed this to detrital silicate grains within samples.

Rare Earth Elements (REEs) can be used as chemical fingerprints that constrain formation environments and conditions. While most REEs are well incorporated in Ca-carbonates, Light REEs (LREE) are rejected more effectively than Heavy REEs (HREE) by magnesite (and siderite) (Bau & Möller, 1992). This is due to the differences in bonding environments, charge, and ionic radii of Ca^2+^, Mg^2+^, and REE^3+^. This is explained in part by lanthanide contraction and the fact that the ionic radius of Ca^2+^ is greater than that of Mg^2+^ in carbonates. Importantly for geochronological considerations, this implies that Sm will be more easily incorporated in to magnesite than Nd motivating the use of a ^147^Sm-^143^Nd isotope chronometer (see Section 6). REE abundances in sedimentary magnesite may signal formation via diagenetic alteration of dolomite rather than direct precipitation prior to burial (e.g., Ellmies et al., 1999; Fernández-Nieto et al., 2003; Lugli et al., 2002; Morteani et al., 1982). For example, Fernández-Nieto et al. (2003) demonstrated that magnesites intermixed with dolomites have lower LREE but similar HREE when compared with the dolomite. Using mass versus concentration REE profiles, they inferred that alteration processes led to the removal of the LREEs while simultaneously exhibiting little influence on the HREEs of the carbonate.

## 6. Absolute Age Dating of Magnesium Carbonate Formation

Given the importance of magnesium carbonates to deciphering ancient aqueous, atmospheric, geologic, and potentially microbial processes in Jezero crater, determining their age and exposure history will help place Jezero within the context of the larger history of Mars. Techniques previously employed on Earth include those with the ability to assess the formation age of magnesite precipitated on the order of millions to billions of years ago (the ^147^Sm-^143^Nd, U-Pb concordia, and ^207^Pb-^206^Pb isochron methods) and within tens of thousands to the last million years (^14^C and U-series disequilibrium dating). There are also techniques that have not been applied to magnesite on Earth but may have the ability to interrogate the surface exposure history of magnesite outcrops (^3^He, ^21^Ne, ^22^Ne, and possibly ^36^Ar).

### 6.1. Techniques for Age Dating Ancient Magnesite

U-Pb and Pb-Pb methods were successfully used to date sparry magnesite to ~1,370–1,380 Ma (Ovchinnikova et al., 2014) and 3,043 ± 59 Ma (Toulkeridis et al., 2010) within the Satka Fm, Russia, and Barberton Greenstone Belt, South Africa, respectively. Sm-Nd isochron ages of between 236 ± 16 Ma and 193.5 ± 8.6 Ma (Henjes-Kunst et al., 2014) were found for sparry magnesite within the Breitenau deposit, Eastern Alps. Magnesite dating using these methods may be challenging due to low parent isotope abundances and/or low parent/daughter isotope ratios. For example, Sm and Nd concentrations in the Breitenau deposit range between 1 ppm and a few hundred ppm, with ^147^Sm/^144^Nd ratios spanning 0.1–0.5 (Henjes-Kunst et al., 2014). It is unknown whether magnesites formed in other environments could be successfully leached to obtain such a favorably large range in Sm/Nd ratio or if they generally host Sm and Nd concentrations adequate for precise geochronology. Sm and Nd concentrations in magnesite samples from the Budd ultramafic complex are at sub-ppm levels (Toulkeridis et al., 2010); similar Sm and Nd concentrations in Martian Mg-rich carbonates would likely preclude successful application of the Sm-Nd method. While sedimentary crystalline magnesite from the Satka Formation exhibits relatively high U concentrations (0.94–2.1 ppm; Ovchinnikova et al., 2014), U concentrations in Mg-rich carbonate veins from the Semail Ophiolite have orders of magnitude lower (0.03–14.8 ppb; Mervine et al., 2015). If U concentrations in magnesium carbonates from Jezero crater are similar to those of the Semail Ophiolite, the application of the isochron technique (e.g., Ovchinnikova et al., 2014; Toulkeridis et al., 2010) may not be feasible for Martian samples. Additionally, mixing of two homogenous sources can lead to the appearance of a ^207^Pb/^204^Pb-^206^Pb/^204^Pb diagram as an isochron. Caution must therefore be taken when employing such a technique; the U-Pb concordia technique is less susceptible to such issues.

The optimal situation for age dating of ancient Jezero carbonates would be lake- or ground-water precipitated carbonates that formed in a single episode with no signs of resetting by post-formation events. Postformation events such as heating associated with meteorite impacts or fluid interactions could affect isotopic systems such that a date would be a recrystallization age. Samples should be examined both texturally and geochemically for evidence of impact events or postformation fluid interactions that have the potential to induce isotopic contamination or entirely reset the U-Pb and Sm-Nd isotope systems. Application of clumped isotope thermometry can shed light on temperatures reached during crystallization or post-formation heating. Care must be taken to determine whether a formation or partial/full recrystallization age is recorded, each of which can constrain the age of magnesium carbonate-forming environments and subsequent geological processes.

### 6.2. Young Veins in Old Ultramafic Rocks

Radiocarbon dating is commonly employed on Earth for systems with carbon-bearing materials with <50 Ka ages. Due to their short half-life, radiocarbon techniques are not likely to be applicable to Martian magnesium carbonates samples. However, continued studies on radiocarbon dating of terrestrial magnesium carbonates could aid in further understanding magnesium carbonate formation in general. Before young radiocarbon dates were reported by Kelemen and Matter (2008), magnesium carbonate veins associated with the Semail Ophiolite were thought to have formed >60 Ma during its emplacement in the Late Cretaceous to Early Tertiary (e.g., Nasir et al., 2007). Such putative antiquity was contradicted by the discovery of magnesite containing live ^14^C and dates spanning ~8–45 Ka (e.g., Kelemen & Matter, 2008; Kelemen et al., 2011; Mervine et al., 2014, 2015). Mervine et al. (2015) confirmed these quaternary formation ages with U-series disequilibrium dating. Radiocarbon in magnesium carbonates can also be used to calculate carbonation rates (Oskierski et al., 2013) and tracing atmospheric carbon sources (Power et al., 2019). Hence, continued studies of radiocarbon within terrestrial magnesium carbonates are warranted in order to understand the degree to which atmospheric carbon and weathering processes play a role in magnesium carbonate formation on Earth. This is an important consideration for Mars because we hope to measure ancient Martian atmospheric conditions through returned magnesium carbonates.

Care must be taken when performing radiocarbon dating of magnesium carbonate because of the many potential sources of carbon in its formation (e.g., Kelemen & Matter, 2008; Kelemen et al., 2011; Mervine et al., 2014; Oskierski et al., 2013; Power et al., 2019). Any ^14^C dates in magnesite must be based on the assumption that carbon incorporated during crystallization retained atmospheric carbon isotope abundances. However, partial exchange of carbon between modern ^14^C-rich fluids and ancient ^14^C-dead carbonate can occur (Mervine et al., 2014). Resolving the extent of C exchangeability between magnesite and fluids is particularly important for porous microcrystalline magnesite.

### 6.3. Surface Exposure Ages

Measurements of cosmogenic isotopes could complement traditional geochronologic measurements focused on the formation age of magnesite. These isotopes are produced via the interaction of galactic cosmic rays with the nuclei that comprise the rocks within the upper 2–3 m of the surface of a planetary body (generally through spallation or neutron capture; Lal, 1988). Based on the chemistry of a sample of interest, the production rate of a given cosmogenic isotope may be derived and a length of exposure calculated (Lal, 1988). On Earth, measurement of radioactive ^10^Be, ^26^Al, and/or ^36^Cl for this purpose is common (e.g., Kubik et al., 1984; Nishiizumi et al., 1986; Phillips et al., 1986; Yiou et al., 1984). On Mars, the duration of exposure is likely to be many millions of years (Farley et al., 2014; Martin et al., 2021; Vasconcelos et al., 2016), such that these radioactive nuclides will be in equilibrium (i.e., produced at the same rate that they decay). As a result, the daughter products of these nuclides must instead be measured to calculate exposure age, if they are uniquely identifiable. Of these, magnesium carbonates lack a target element for production of ^26^Al, precluding its use. ^10^Be decays to ^10^B, which is the common isotope of boron and therefore cannot reliably be measured above background levels. The main daughter product of ^36^Cl (produced by neutron capture on ^35^Cl) is ^36^Ar, a noble gas which is otherwise unlikely to be present in the crystal structure, making this a potentially useful exposure chronometer if it is produced in measurable quantities (discussed further below).

The spallogenic noble gases ^3^He, ^21^Ne, and ^22^Ne are stable and likely to be produced at relatively high rates due to the large proportion of target Mg and (for ^3^He) O in magnesium carbonate (Wieler, 2002). Noble gases are highly insoluble in crystalline material and will therefore be lost by diffusion if permitted by the thermal history and kinetic parameters of the material of interest. This low solubility also means that the noble gas contents of a given sample will be reset if recrystallization occurs. For cosmogenic dating of Martian magnesite, the primary determination is therefore whether a given noble gas is effectively trapped in the magnesite crystal structure at Martian surface temperatures, and whether evidence for recrystallization is present. No formal studies of the diffusion kinetics of magnesite have been performed with any of ^3^He, ^21^Ne, ^22^Ne, and ^36^Ar. Therefore, we turn to studies that have been performed on other carbonate minerals as a proxy for noble gas behavior in magnesite, recognizing that these crystal systems are not identical.

A study of ^4^He diffusion in calcite and dolomite found that below ~50°C, helium is likely to be retained in these carbonate minerals (Copeland et al., 2007). However, attempts at applying (U-Th)/He dating in natural calcite samples have been met with mixed success (Copeland et al., 2007; Cros et al., 2014), likely due to complex multidomain diffusion kinetics inherent to calcite (Amidon et al., 2015; Copeland et al., 2007). A subsequent study attempted calcite ^3^He exposure measurements directly and similarly found reliable results in only some cases (Amidon et al., 2015). Promisingly, calcite minerals with a higher Mg wt% demonstrated more robust He retention in this study (Amidon et al., 2015), suggesting that magnesite may function more reliably as a He geochronometer than calcite.

Neon diffusivity in carbonate minerals has not been assessed. However, an empirical anticorrelation between atomic radius and diffusivity has been observed in a range of minerals (Baxter, 2010), suggesting that as an extremely rough approximation, the diffusivity of Ne in carbonates should lie somewhere between that of He and Ar. Given the closure temperatures of ~50°C for He (Copeland et al., 2007) and ~385°C for Ar (Cassata & Renne, 2013), this approximation hints that ^21^Ne and ^22^Ne may be suitable for cosmogenic dating applications in Martian magnesium carbonates. ^21^Ne and ^22^Ne are likely to be produced at relatively high rates in magnesium carbonates in this case, because Mg is a major target element for the production of ^21^Ne and ^22^Ne via spallation (Wieler, 2002). We therefore assess ^21^Ne and ^22^Ne exposure dating to be a promising method of determining the length of magnesite exposure on Mars.

The only study of argon diffusivity in calcite suggests a closure temperature of 385 ± 2°C (Cassata & Renne, 2013), indicating that this noble gas could be quantitatively retained in magnesium carbonate minerals at Martian surface temperatures. However, it is uncertain whether significant amounts of ^36^Ar would be produced in such minerals. The major component elements of magnesium carbonates (Mg, C, O) are all below that of ^36^Ar, precluding spallogenic production. The sole mechanism of ^36^Ar production in magnesium carbonates is therefore through decay of ^36^Cl, which is produced via neutron capture of ^35^Cl, so inclusion of trace chlorine or chloride contaminants is key for the success of a ^36^Ar approach. Elevated Cl concentrations are observed in rocks of Meridiani planum and Gale crater where evaporative processes were involved (Clark et al., 2005; Thomas et al., 2019; Tosca et al., 2005). However, chlorine does not partition readily into calcite, aragonite, or dolomite (natural and synthetic samples generally contain <0.01 wt% Cl; Kitano et al., 1975; Staudt et al., 1993), casting doubt on the viability of this method unless abnormally high Cl concentrations or inclusions of minerals such as halite are observed in Martian magnesium carbonates.

## 7. Summary and Implications for the 2020 Rover Mission

Based on understanding from current orbital data, the Mars 2020 Perseverance rover will encounter magnesite or its hydrous relatives at the Jezero landing site on Mars, associated with olivine-enriched units. By comparison with examples on the Earth, we expect to observe magnesium carbonate in at least one of the five distinct expressions that include the following: (a) Precipitation in veins hosted in ultramafic-rocks formed by circulation of meteoric fluids or deeper hydrothermal fluids through fracture networks; (b) within the matrix of carbonated ultramafic rocks from hydrothermal alteration; (c) Nodule formation as an evaporative soil process; (d) Precipitation as authigenic sediment in alkaline lakes and playas; and, (e) Diagenetic replacement of precursor carbonates such as dolomite, calcite, and hydrous magnesium carbonates. The formation environments of the first four magnesium carbonate types span a spatial hydrologic gradient from uplifted ultramafic rocks to down-gradient sedimentary basin and may provide a close analog for the magnesium carbonates in the Jezero crater region.

The most commonly preserved magnesium carbonate in terrestrial environments is magnesite. Hydrous magnesium carbonates typically cooccur with magnesite in alkaline lakes, playa, and soil environments. This observation and the pathways to formation of magnesite in general remains an enigma from a thermodynamic and kinetics perspective. Magnesite is more soluble than its Ca-rich counterparts and precipitation occurs slowly due to kinetic inhibition by solvated Mg ions. Hydrous magnesium carbonates are less kinetically inhibited and may subsequently transform to magnesite. Understanding the thermodynamic stability of magnesite as a primary precipitate and as a recrystallization product will therefore be important for constraining physical conditions for magnesite and potentially hydrous magnesium carbonate formation on Mars.

Diagnostic textures and mineral assemblages can be directly analyzed with the Perseverance rover and record processes of formation and may also preserve biologic fabrics, assuming that life appeared or originated and was widely dispersed on Mars. Textures and phases associated with mineral precipitation include the following: (a) Fine-grained or microcrystalline vein- or matrix-forming magnesium carbonates associated with ultramafic terrains and carbonation products; (b) Nodules and bladed aggregates found in soils situated near ultramafic terrains; (c) Microcrystalline magnesium carbonates precipitated as primary crusts in playas and alkaline lakes; (d) Microcrystalline carbonates that replace limestones and dolostones; (e) Sparry calcite that form from metasomatic and metamorphic replacement. Thrombolites, stromatolites, crinkly and pustular laminites, botryoidal or spherulitic textures, and microscale filament, spheroids, and peloids may yield insight into whether or not biologic processes operated on the surface of ancient Mars if returned to Earth and studied in the laboratory. If life never originated on Mars, sampling these textures and fabrics could still provide significant insight into past environments and the history of water. The rover contains a suite of instruments that can detect and discriminate magnesium carbonate phases and then once confirmed, it moves to more detailed analyses of these textures and mineral assemblages to determine their relation to ultramafic terrains, sedimentary, or diagenetic environments, providing insights into the evolution of ancient Mars and informing carbonate sampling strategies.

Establishing the δ^13^C and δ^18^O compositions of the surface-atmospheric environment at the time of magnesium carbonate formation will significantly aid in further understanding the carbon cycle on ancient Mars. This is due to the limited record of previous isotopic measurements of Martian surface materials. Aside from measurements of carbonate globules within the ALH84001 meteorite, the Jezero carbonates will yield the first insight into the δ^13^C and δ^18^O composition of the ancient Martian surface-atmospheric environment, providing invaluable new data points for the isotopic evolution of the Martian atmosphere, surface, and potentially the subsurface. Furthermore, stable isotope ratios of carbon, oxygen, and magnesium in magnesium carbonate minerals have proven highly valuable proxies of conditions (climate, fluid and atmospheric chemistry, surface or subsurface alteration processes, and biospheric evolution) in the Earth’s past and can be applied similarly for Martian samples (e.g., Zachos et al., 2001). Clumped isotope methodologies and fluid inclusion thermometry for magnesium carbonates are promising for establishing temperature and equilibrium/disequilibrium conditions of formation but are still in development. A number of radiometric dating techniques, including ^147^Sm-^143^Nd isochron dating, U-Pb concordia, and ^207^Pb-^206^Pb isochron dating have yielded promising results for magnesites on Earth and could potentially be utilized for dating Martian magnesium carbonate samples. In addition, cosmogenic exposure dating utilizing the noble gases ^3^He, ^21^Ne, ^22^Ne, or ^36^Ar could potentially be applied to returned Martian magnesium carbonate samples but it has not yet been attempted for terrestrial samples. Continued radiocarbon and U-series disequilibrium dating of young terrestrial samples may yield further insight into weathering processes and the role of atmospheric carbon in magnesium carbonate formation.

As we prepare for analyzing magnesium carbonates returned by the Mars 2020 mission, there are several notable areas in laboratory experiments given below that we can work to improve: (a) Understanding of the ability of magnesium carbonates to preserve biosignatures. (b) Understanding of the thermodynamic, kinetic, or metabolic conditions needed to induce precipitation, transformation, and replacement of various magnesium carbonates. (c) Understanding of tracking sources and transport of carbon, magnesium and fluids through isotope systematics, specifically focusing on understanding the origin of the large range in δ^13^C and δ^18^O signatures and how these reflect sources, aqueous conditions, and/or disequilibrium conditions in different environmental settings; (d) Methodology for performing clumped isotope and fluid inclusion thermometry of magnesium carbonates; (e) Focus on performing more radiogenic and cosmogenic dating experiments on magnesium carbonates to narrow down the most appropriate techniques applicable to ancient Martian magnesium carbonates.

## Supplementary Material

Supplement

## Figures and Tables

**Table 1:** Overview of all Characterized Magnesium Carbonate Textures, Their Associated Paragenetic Mineral Assemblages and Geological Setting as Described in Section 3

Texture/crystal habit	Paragenetic mineral assemblage	Geological setting	Fig
Fine-grained or microcrystalline veins	magnesite ± hydromagnesite ± talc ± silica ± olivine ± serpentine ± dolomite ± calcite	Ultramafic terrains	3
Fine-grained or microcrystalline matrix of carbonated ultramafic rock	magnesite ± hydromagnesite ± talc ± silica ± olivine ± serpentine ± brucite ± quartz ± dolomite ± calcite	Ultramafic terrains	3
Nodules and bladed aggregates	magnesite ± hydromagnesite ± nesquehonite ± dypingite ± silica	Pedogenic soils	3
Fine-grained or microcrystalline mounds	magnesite ± hydromagnesite ± nesquehonite ± dypingite ± aragonite ± sulfates ± halite	Hypersaline lakes and playas	4
Thrombolites, nodules, stromatolites/laminated lithified mats, botryoids	magnesite ± hydromagnesite ± Mg-silicates	Alkaline lakes	4
Spherulites/coccoids	magnesite ± hydromagnesite ± dolomite	Alkaline lakes	4
Coatings on filaments and detrital grains	magnesite ± hydromagnesite	Alkaline lakes	4
Microcrystalline	magnesite ± calcite ± dolomite	Diagenetic replacement	5
Spar	magnesite ± tremolite, quartz ± calcite ± talc ± diopside	Metasomatic replacement	5
Spar	magnesite ± garnet ± diopside in peridotite or eclogite assemblages	Ultra-high pressure (UHP) terrains	5

*Note*. Right column shows accompanying figure number with detailed textural images at micro-, meso-, and macro-scales.

## Data Availability

All recognition of reused images should be given to original authors with references that are denoted on each figure. Spectral data from Figure 1 can be found in Mandon et al. (2020) and Ehlmann et al. (2009). Isotopic data from Figure 6 is a compilation of data color coded according to references as given in the figure legend. A table of data has been uploaded to the Caltech Data Repository: 10.22002/D1.1971.

## Key Points:

- On Earth, magnesium carbonates of distinct textures form in ultramafic rock hosted veins, listvenites, soils, alkaline lakes, and playas
- The isotopic and elemental composition of terrestrial magnesium carbonate reveal timing and chemistry of fluid conditions
- Through the Mars 2020 sample return mission, the reviewed methods can be used to analyze carbonate-forming conditions on ancient Mars
